# Circulating vitamin D status and prognosis in colorectal cancer: a systematic review and meta-analysis with exploratory evidence on vitamin D receptor polymorphisms

**DOI:** 10.1186/s12885-026-16026-x

**Published:** 2026-04-16

**Authors:** Daylia Thet, Nutthada Areepium, Chidchanok Rungruang, Nattawut Leelakanok, Tippawan Siritientong

**Affiliations:** 1 Department of Food and Pharmaceutical Chemistry, Faculty of Pharmaceutical Sciences, Chulalongkorn University, Phyathai Road, Pathumwan, Bangkok, 10330 Thailand; 2 Department of Pharmacy Practice, Faculty of Pharmaceutical Sciences, Chulalongkorn University, Bangkok, 10330 Thailand; 3 Division of Clinical Pharmacy, Faculty of Pharmaceutical Sciences, Burapha University, Chonburi, 20131 Thailand; 4 Center of Excellence in Metabolomics for Life Sciences, Chulalongkorn University, Bangkok, 10330 Thailand

**Keywords:** Vitamin D, Colorectal cancer, Prognosis, Prognostic biomarker, Survival, Vitamin D receptor gene polymorphism

## Abstract

**Background:**

Circulating 25-hydroxyvitamin D [25(OH)D] levels and genetic polymorphisms in the vitamin D receptor (VDR) have been explored as potential prognostic factors in colorectal cancer (CRC). This study aimed to investigate the association between circulating 25(OH)D levels and CRC prognostic outcomes, with a narrative evaluation of VDR polymorphisms.

**Methods:**

We performed a systematic literature search in the Cochrane Library, PubMed, ScienceDirect, and Scopus. The primary outcomes were CRC-specific survival, overall survival (OS) and disease-free survival (DFS). Hazard ratios (HRs) with 95% confidence intervals (CIs) were pooled using the random-effects model with inverse variance weighting, comparing high versus low 25(OH)D levels. Due to heterogeneity in genetic models and limited available data, VDR polymorphisms were synthesized using a narrative approach.

**Results:**

Of the 61 studies included in the systematic review, 35 studies were included in the meta-analysis, which showed that higher 25(OH)D levels were associated with a lower risk of mortality, including a 26% lower CRC-specific mortality (HR 0.74; 95% CI 0.69–0.80; I^2^ = 0.01%), a 32% lower overall mortality (HR 0.68; 95% CI 0.64–0.72; I^2^ = 7.6%), and improved DFS (HR 0.71; 95% CI 0.61–0.83; I^2^ = 36.8%). The prognostic roles of VDR polymorphisms, particularly rs7975232 (*ApaI*), rs1544410 (*BsmI*), rs2228570 / rs10735810 (*FokI*), and rs731236 (*TaqI*) with CRC outcomes, including CRC-specific survival, OS, and DFS were reported across 18 studies.

**Conclusion:**

This study provides quantitative evidence supporting the potential prognostic relevance of circulating vitamin D levels in CRC. The role of VDR genetic polymorphisms remains inconclusive, warranting further investigations.

**Trial registration:**

The protocol of this systematic review and meta-analysis has been registered on PROSPERO (https://www.crd.york.ac.uk/PROSPERO/), with the registration number CRD42024575841.

**Supplementary Information:**

The online version contains supplementary material available at 10.1186/s12885-026-16026-x.

## Introduction

Colorectal cancer (CRC) is a common malignancy with a high incidence globally, with up to 1.6 million deaths projected by 2040 [1]. CRC carcinogenesis results from complex interactions between genetic susceptibility and environmental exposures, among which diet represents a significant modifiable risk factor. Epidemiological evidence has demonstrated that dietary patterns characterized by high consumption of red and processed meats and low intake of fiber and calcium are associated with an increased risk of CRC [2, 3]. Emerging research further highlights the influence of specific dietary components such as dietary acid load on CRC progression and metastatic risk, with higher potential renal acid load being associated with an increased risk of metastatic CRC [4]. In the prognosis and pathogenesis of CRC, the clinically important role of vitamin D has been increasingly investigated. Vitamin D status, often assessed through serum 25-hydroxyvitamin D [25(OH)D] levels, has also been examined in relation to CRC outcomes. Higher circulating 25(OH)D levels have been associated with reduced CRC risk and improved prognosis in patients with CRC [5]. Prospective studies have demonstrated an inverse relationship between plasma 25(OH)D levels and mortality, with individuals in the highest quartile of circulating vitamin D exhibiting better survival outcomes compared to those in the lowest quartile [6]. Additionally, low vitamin D levels have been associated with adverse clinical outcomes in patients with CRC, including an increased risk of recurrence, accelerated tumor progression, and a higher incidence of treatment-related complications [7].

The biologically active form of vitamin D, 1,25-dihydroxycholecalciferol, is synthesized through hydroxylation by the enzyme 1-alpha hydroxylase. Upon activation, vitamin D may regulate apoptosis, cellular differentiation, and proliferation in cancer cells by binding to the vitamin D receptor (VDR) [8]. VDR, a nuclear receptor encoded on chromosome 12q, functions as a transcription factor that modulates the expression of genes involved in oncogenesis. Variations in the VDR gene, including single nucleotide polymorphisms (SNPs), can alter mRNA stability, protein translation, and the functionality of the VDR protein, potentially impacting the biological pathways critical to CRC development and progression [9]. Among the numerous SNPs identified within the VDR gene, the most extensively studied and clinically significant variants include *ApaI*, *BsmI*,* FokI*, and *TaqI* [10, 11]. Previous reports have highlighted the association between VDR polymorphisms and CRC risk. However, findings regarding specific genotypes and their roles in CRC susceptibility have varied across studies [12–15]. Beyond their impact on CRC incidence, the influence of VDR polymorphisms on prognostic outcomes, particularly survival, has gained increasing attention in recent years [16–18].

Vitamin D has both genomic effects through VDR and non-genomic effects through rapid signaling pathways that use membrane-bound receptors such as PDIA3 (also known as 1,25D-MARRS). These pathways can modulate intracellular calcium, oxidative stress, and apoptotic signaling pathways, which may help regulate tumors [19]. These multi-pathway mechanisms further support the role of vitamin D and its analogues in CRC prevention and treatment, particularly as adjuncts to cytostatic agents, and highlight the potential utility of gene expression profiling of key regulators like VDR for optimizing treatment strategies [20]. Recent studies suggest that vitamin D influences key elements of the tumor microenvironment (TME), such as immune cell infiltration, angiogenesis, and the epithelial-mesenchymal transition (EMT), which play crucial roles in CRC progression and metastasis [21]. Although laboratory and population-based research highlights its therapeutic ability, especially via modulation of the TME, the lack of well-designed clinical trials and unclear optimal dosing limit its integration into routine clinical practice. A transcriptomic study using CRC cell lines and patient-derived organoids showed that calcitriol affects important genes involved in tumorigenesis such as CDH1, GADD45, EFTUD1, and KIAA1199. This gene regulation supports the role of vitamin D in controlling tumor progression and suggests its potential as a therapeutic agent [22].

Recent evidence suggests that VDR may influence CRC progression by directly regulating carcinogenic pathways, particularly Wnt/β-catenin. VDR interacts with β-catenin, suppresses its nuclear accumulation, and downstream targets such as cyclin D1 and lymphoid enhancer factor (LEF‑1), thereby inhibiting cancer cell invasion and promoting apoptosis [23]. Given the growing interest in exploring the pharmacogenomic implications of VDR SNPs in cancer susceptibility and outcomes [24], integrating vitamin D biomarkers with genomic data may enhance cancer risk prediction and prognostic assessments. As oncology research increasingly focuses on inter-individual variability, identifying individuals at risk of poor clinical outcomes can be supported by incorporating both modifiable biomarkers such as vitamin D and stable genetic markers such as VDR SNPs. Therefore, this study aims to systematically review the association between circulating 25(OH)D levels and prognostic outcomes in CRC, with a complementary narrative evaluation of VDR gene polymorphisms. We focus on survival outcomes, tumor progression, and recurrence, providing a systematic overview of the potential clinical relevance.

## Materials and methods

This systematic review and meta-analysis was reported in accordance with the Preferred Reporting Items for Systematic Reviews and Meta-Analyses (PRISMA) guidelines [25]. The completed PRISMA checklist is provided in Supplementary Table S1.

### Protocol registration

The study protocol was approved for exemption from Ethics Review by the Research Ethics Review Committee for Research Involving Human Research Participants, Group I, Chulalongkorn University (COA No. 190/67), in accordance with the Declaration of Helsinki. It was also registered in PROSPERO, an international database of prospectively registered systematic reviews (CRD42024575841).

### Selection criteria

PICO criteria for the selection of the included studies are described in Table 1. Full-text articles that assessed circulating 25(OH)D concentration or VDR polymorphisms, including rs7975232 (*ApaI*), rs1544410 (*BsmI*), rs2228570 (also known as rs10735810; *FokI*), and rs731236 (*TaqI*) in CRC patients, and reported CRC prognostic outcomes published in English were included. Studies involving oral vitamin D intervention were excluded, as the objective of this systematic review was to assess the prognostic associations of endogenous 25(OH)D levels and VDR genetic variations rather than the therapeutic effects of vitamin D intervention. The inclusion of intervention trials could introduce clinical heterogeneity related to dosage, duration, and treatment protocols, which fall outside the scope of the present study. Studies focusing on other cancer types, conference abstracts or posters, study protocols, books, editorials, guidelines, letters, in vitro studies, in vivo or animal studies, and review articles were also excluded.

**Table 1:** PICO criteria for the study selection

Population	Patients diagnosed with colorectal cancer
Exposure	Serum vitamin D measurement, VDR polymorphism analysis
Comparison	Vitamin D (high vs. low) groups, VDR genotypes
Outcome	Survival, prognostic outcomes

### Literature search

The literature search was conducted according to the PRISMA guidelines [25]. Four databases: Cochrane Library, PubMed, ScienceDirect, and Scopus were searched from the inception of each database to February 2026. EMBASE and Web of Science were not included due to institutional access limitations. Search terms included primary exposures (vitamin D receptor gene polymorphism, 25-hydroxyvitamin D, calcidiol, serum vitamin D) and colorectal cancer. Additionally, the reference lists of records from databases were searched. Full search strategies are available in Supplementary Table S2.

### Data extraction

The search results were exported to the citation manager (EndNote 20.6, Clarivate Analytics, New York, NY, USA). After removing duplicates, titles and abstracts were thoroughly screened. Eligible records were independently selected by two authors for inclusion in this systematic review, while reasons for exclusion (no CRC, no VDR, no vitamin D measurements, or review articles) were recorded. After screening full-text articles, data extraction and quality assessment were performed by two independent authors. Information on author, year of publication, study design, study setting, characteristics of CRC patients, sample size, any reported CRC treatments (chemotherapy or targeted therapy, radiotherapy, or surgery), rs numbers of VDR polymorphisms, and prognostic outcomes including CRC-specific survival, overall survival (OS), disease-free survival (DFS), recurrence-free survival (RFS), progression-free survival (PFS), and any reported side effects was extracted. Discrepancies were resolved by discussing with a third author. In addition, the data entry was judged by the co-authors. Any disagreements were resolved by consensus among all authors.

OS was defined as the time from diagnosis or treatment initiation to death from any cause. CRC-specific survival was defined as the time to death attributable to CRC. RFS was defined as the time to first recurrence of CRC or death from any cause. DFS was defined as the time to recurrence of disease, occurrence of a second primary CRC, or death from any cause. TTR was defined as the time to first documented recurrence of CRC, with deaths without documented recurrence treated as censored events. PFS was defined as the time to disease progression or death from any cause. These definitions reflect commonly used criteria; however, specific definitions may vary slightly across the included studies.

Studies reported 25(OH)D levels as low or high based on quartiles, tertiles, quantiles, or predefined categories. 25(OH)D concentrations were measured in ng/mL or nmol/L, with thresholds varying across studies. Some studies defined low levels using cutoffs as low as < 6.2 ng/mL, while others classified levels > 30 ng/mL as adequate. Several studies grouped participants into categories such as sufficient, insufficient, or deficient rather than strictly as low or high. These variations were addressed by standardizing comparisons as high and low groups within the context of each study’s reporting framework. However, since there were differences in cutoff values and categorization strategies across studies, pooled estimates should be interpreted with caution.

### Quality assessment

The methodological quality assessment of the included cohort studies was performed using the Newcastle-Ottawa Quality Assessment Scale [26, 27]. For cohort studies, information on the representativeness of the exposed cohort, selection of the non-exposed cohort, ascertainment of exposure, reporting about the outcome was not present at the beginning of the study, comparability of cohorts, assessment of the outcome, follow-up duration, adequacy of follow-up cohorts was evaluated. For case-control studies, we evaluated the adequate case definition, representativeness of the cases, selection of controls, the definition of controls, comparability of cases and controls, ascertainment of exposure, ascertainment of method, and non-response rate. Domains including representativeness of the sample, sample size, non-respondents, controlled confounders, assessment of the outcome, and statistical tests were evaluated for cross-sectional studies (Supplementary Table S3). The certainty of evidence for each survival outcome was evaluated using the Grading of Recommendations Assessment, Development and Evaluation (GRADE) framework for prognostic factor studies (Supplementary Table S4). Evidence was assessed across five domains: risk of bias, imprecision, inconsistency, indirectness, and publication bias. The certainty of evidence was categorized as high, moderate, low, or very low [28].

### Statistical analysis

The statistical analyses of quantitative data were performed using R software (R Core Team, 2025), and the metafor package was used for meta-analysis. The hazard ratios (HRs) with 95% confidence intervals (CIs) were extracted from multivariate analyses of survival outcomes. To standardize HRs across studies with differing reference groups, HRs were inverted if necessary by calculating the reciprocal of the HR and reversing the corresponding 95% CI limits (i.e., lower limit = 1/upper, upper limit = 1/lower), ensuring consistent comparisons of the outcomes between high and low levels of vitamin D groups. Effect estimates were pooled on the logarithmic scale using inverse-variance weighting. This transformation was performed to maintain a uniform interpretation of survival outcomes in the meta-analysis. Pooled effect estimates were calculated using random-effects models with the restricted maximum likelihood (REML) estimator to account for potential between-study heterogeneity. The I^2^ statistic was used to assess heterogeneity among the included studies (> 75% indicating high heterogeneity and < 25% indicating low heterogeneity) [29]. To explore the potential sources of heterogeneity, subgroup analyses were conducted according to disease stage (metastatic CRC and stage III CRC) and the timing of vitamin D measurement (pre-diagnostic vs. post-diagnostic). The pooled results represent adjusted estimates to account for confounding factors reported in the original studies. The 95% prediction intervals were calculated. Where feasible, subgroup or sensitivity analyses were considered to explore the impact of differences in vitamin D category definitions across studies. Additionally, sensitivity analyses were performed by excluding studies rated as low quality for each outcome where applicable. A sensitivity analysis was conducted including only studies using harmonized clinical thresholds for vitamin D status, defined as sufficient (≥ 50 nmol/L or ≥ 20 ng/mL) versus deficient (< 30 nmol/L or < 12 ng/mL). Publication bias was assessed by visual inspection of the funnel plot and Egger’s regression test. Statistical significance was set at *P* < 0.05.

## Results

### Study characteristics

**Fig. 1:**
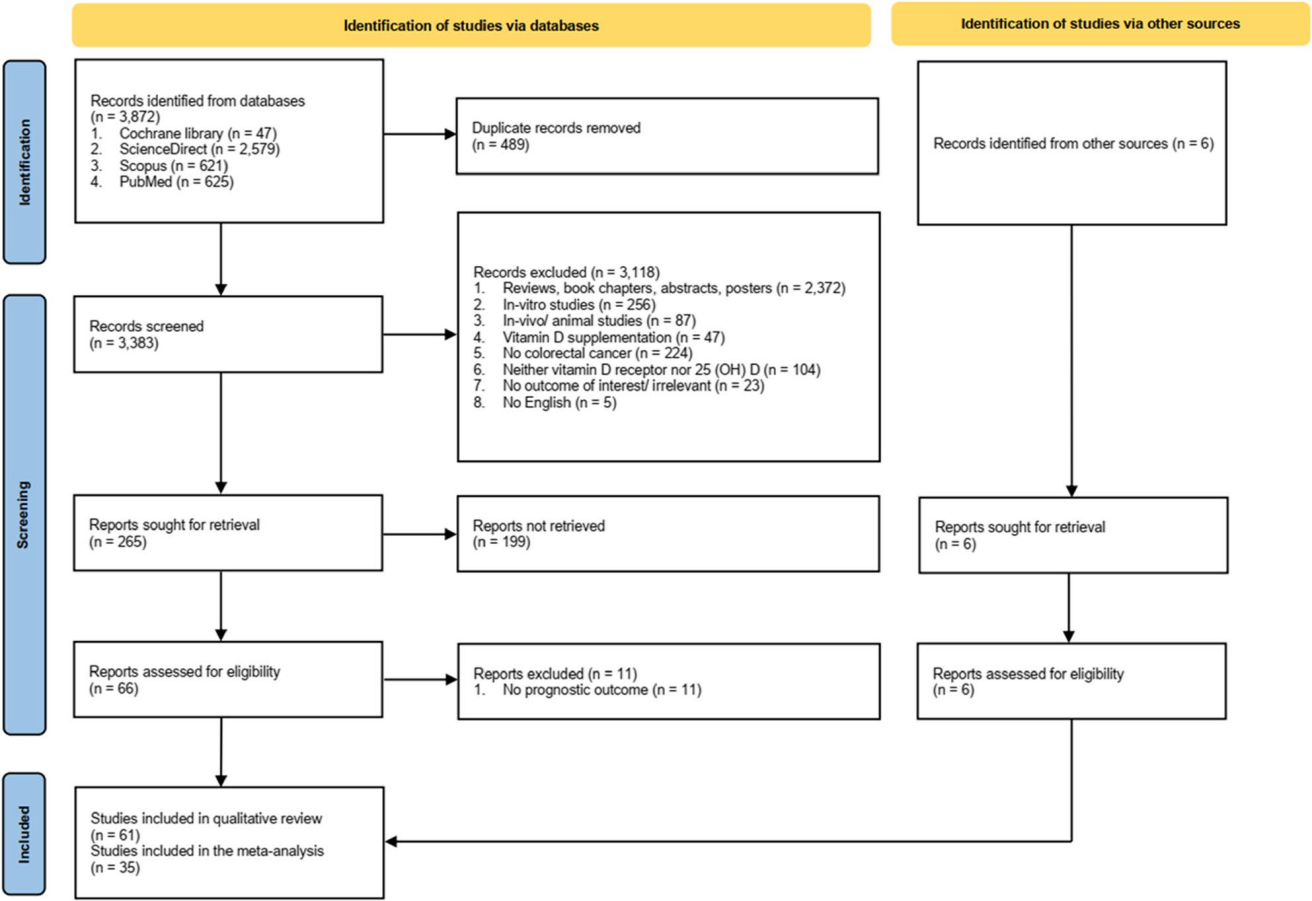
PRISMA flow diagram of study selection process

A total of 3,872 records were retrieved from the database search; of these, 489 records were identified as duplicates. Six articles were retrieved from manual searching through reference lists, additionally. After screening titles and abstracts, followed by full-text readings, 72 articles remained for potential inclusion in the qualitative synthesis. However, in 11 records, no prognostic outcomes in CRC patients were identified. Figure 1 depicts the systematic search of records.

### Studies included in qualitative synthesis

Finally, 61 records were included in the systematic review [6, 16–18, 30–86]. Table 2 provides an integrative summary of the included studies, while detailed characteristics of each study are available in Supplementary Table S5. There were 49 articles reporting 25(OH)D levels [6, 30, 32–36, 38, 40–49, 52, 53, 55–67, 69–71, 73–79, 81–86], 12 articles reporting VDR polymorphisms [16–18, 31, 37, 39, 50, 51, 54, 68, 72, 80], and 6 articles reporting both exposures [34, 40, 59, 64, 77, 81], which are also included in the 25(OH)D studies. Most of the studies were conducted in Western countries (the United States, Germany, the United Kingdom, Greece, Australia, Finland, and Italy). In Asia, there were 3 studies from China, 1 study from South Korea, and 1 study from Japan. Diverse racial and ethnic backgrounds were noted: Black, White, and Asian. Studies included patients at any stage of CRC, only stage III CRC, or specifically metastatic CRC patients. Treatment information was reported in 39 studies, which included surgery, radiotherapy, and/or chemotherapy. Across the studies included in the qualitative systematic review, follow-up duration ranged from 2.2 to 28 years.

**Table 2:** Integrative summary of the included studies

Category	Summary
Study information	Total number of studies (*n* = 61) Publication years: 2007–2024 Regions: Asia (*n* = 5), Western countries (*n* = 56)
Population	Total participants: 55,176 Median sample size: 522 (237–1169)
CRC characteristics	Stage I–III (*n* = 16) Stage I–IV (*n* = 21) mCRC (*n* = 11) Stage not reported (*n* = 13)
Exposures	Blood vitamin D measured by chemiluminescent immunoassay (*n* = 10), enzyme immunoassay (*n* = 3), mass spectrometry-based assay (*n* = 13), mixed (*n* = 1), radioimmunoassay (*n* = 19), unspecified (*n* = 3), High-pressure liquid chromatography with ultraviolet detection (*n* = 1) VDR SNPs: *ApaI* (*n* = 5), *BsmI* (*n* = 5), *FokI* (*n* = 4), *TaqI* (*n* = 4), *Cdx2* (*n* = 4), others (*n* = 3)
CRC treatments	Surgery (*n* = 4) Surgery + Chemotherapy (*n* = 16) Surgery + Chemotherapy + Radiotherapy (*n* = 10) Chemotherapy (*n* = 8) Chemotherapy + Radiotherapy (*n* = 1)
Outcomes^a^	OS (*n* = 46) CSS (*n* = 27) DFS (*n* = 12)

Note: ^a^some studies reported more than one prognostic outcomes

Abbreviations: *CSS* colorectal cancer-specific survival, *DFS* disease-free survival, *mCRC* metastatic colorectal cancer, *OS* overall survival, *SNP* single nucleotide polymorphism, *VDR* vitamin D receptor

There were 55,176 CRC patients in the 61 included studies. Circulating 25(OH)D levels were assessed either pre-diagnostic, post-diagnostic, pre-operative, or post-operative. The 25(OH)D levels were assessed using radioimmunoassay or liquid chromatography-tandem mass spectrometry. Most included genetic studies on VDR polymorphisms and CRC outcomes assessed deviations from Hardy-Weinberg equilibrium (HWE) and used established genotyping techniques such as real-time PCR, TaqMan assays, or sequencing to ensure reliable genetic data. No significant deviations from HWE were reported, suggesting acceptable genotyping quality.

### Association between VDR SNPs and prognostic outcomes in CRC patients

The association between VDR gene polymorphisms and survival outcomes in CRC patients has been widely investigated in several studies, particularly for rs7975232 (*ApaI*) [16, 17, 40, 72, 80], rs1544410 (*BsmI*) [16, 34, 39, 50, 80], rs2228570 (*FokI*) [16, 34, 37, 39, 50], rs731236 (*TaqI*) [16, 18, 37, 50], rs11568820 (*Cdx2*) [37, 40, 77, 81], and other VDR SNPs [51, 54, 68]. However, the findings remain inconsistent across studies regarding CRC-specific survival, OS, DFS, and other outcomes, as summarized in Table 3. Given the limited number of studies for individual VDR SNPs, variability in genetic models, and heterogeneity in outcomes across studies, a quantitative synthesis was precluded, and therefore, the results were presented as a narrative exploratory summary of the prognostic outcomes.

**Table 3:** Association of VDR SNPs with prognostic outcomes in CRC patients

VDR SNPs	Outcomes	Main findings	Ref
rs7975232 (*ApaI*)	OS	*ApaI* homozygous mutant genotypes (HR 1.6; 95% CI 1.3–2.0; *P* < 0.001)	[16]
	DFS	*ApaI* mutant alleles (HR 1.56; 95% CI 1.0–2.3; *P* = 0.031)	[72]
	OS	*ApaI* wild-type alleles (*P* = 0.021)	[80]
	OS	*ApaI*-AA genotype compared to C allele carriers (HR 1.97; 95% CI 1.04–3.73; *P* = 0.036)	[17]
	CSS	*ApaI*-CC genotype (HR 0.28; 95% CI 0.13–0.60; *P* = 0.001); AA genotype (HR 0.43; 95% CI 0.24–0.79; *P* = 0.005)	[40]
rs1544410 (*BsmI*)	CSS	No significant association (*P* = 0.42)	[34]
	OS	No significant association (*P* = 0.644)	[16]
	OS/ DFS	No significant association (*P* > 0.05)	[50]
	OS	*BsmI* wild-type alleles (*P* = 0.033)	[80]
	ColonC-SS	*BsmI*-BB genotype compared to bb carriers (HR 1.50; 95% CI 1.06–2.12; *P* = 0.045)	[39]
rs2228570 (*FokI*)	CSS	No significant association (*P* = 0.90)	[34]
	OS	*FokI* homozygous mutant genotypes (HR 1.4; 95% CI 1.1–1.8; *P* = 0.005)	[16]
	OS/ CSS	No significant association	[37]
	ColonC-SS	No significant association (*P* = 0.079)	[39]
	OS/ DFS	No significant association (*P* = 0.100)	[50]
rs731236 (*TaqI*)	OS	*TaqI*-GG genotype carriers compared to AA or AG carriers of mCRC patients (HR 0.62; 95% CI 0.39–0.99; *P* = 0.046)	[18]
	OS	*TaqI* homozygous mutant genotypes (HR 1.3; 95% CI 1.0–1.6; *P* = 0.029)	[16]
	OS/ CSS	No significant association	[37]
	OS/ DFS	No significant association (*P* > 0.05)	[50]
rs11568820 (*Cdx2*)	OS/ CSS/ DFS/ RFS	No significant association	[77]
	OS/ CSS	No significant association	[37]
	OS/ CSS	No significant association (*P* > 0.05)	[81]
	CSS	*Cdx2*-GG genotype (HR 0.54; 95% CI 0.36–0.79; *P* = 0.001)	[40]
Other VDR SNPs	Grade 3–4 neutropenia	rs12717991 (OR 0.36; 95% CI 0.16–0.82; *P* = 0.015) rs11168287 (OR 3.12; 95% CI 1.02–9.56; *P* = 0.046) rs11574026 (OR 2.36; 95% CI 1.06–5.23; *P* = 0.035)	[51]
	Grade 3–4 GI toxicity	rs11574077 (OR 4.46; 95% CI 1.43–13.96; *P* = 0.010) rs4760648 (OR 2.09; 95% CI 1.13–3.84; *P* = 0.018) rs2853564 (OR 0.38; 95% CI 0.18–0.78; *P* = 0.008)	[51]
	OS	rs7299460 (HR 0.61; 95% CI 0.43–0.88; *P* = 0.0075) in discovery cohort rs7299460 (HR 0.57; 95% CI 0.33–0.99; *P* = 0.0477) in replication cohort	[54]
	PFS	rs7299460 (No significant association, *P* > 0.05)	[54]
	OS	rs7299460 CC genotype compared to the CT/TT genotypes (HR 1.53; 95% CI 1.15–2.03)	[68]

Abbreviations: *ColonC-SS* colon cancer-specific survival, *CSS* colorectal cancer-specific survival, *DFS* disease-free survival, *GI* gastrointestinal, *mCRC* metastatic colorectal cancer, *OS* overall survival, *PFS* progression-free survival, *RFS* recurrence-free survival, *SNP* single nucleotide polymorphism, *VDR* vitamin D receptor

### Meta-analysis results

We conducted quantitative syntheses to evaluate the associations between circulating 25(OH)D levels and survival outcomes in patients with CRC. A detailed summary of study-specific vitamin D exposure definitions, timing of measurement, outcomes, and effect estimates used in the meta-analysis is provided in Supplementary Table S6.

### Association of circulating 25(OH)D levels with CRC-specific survival and overall survival

**Fig. 2:**
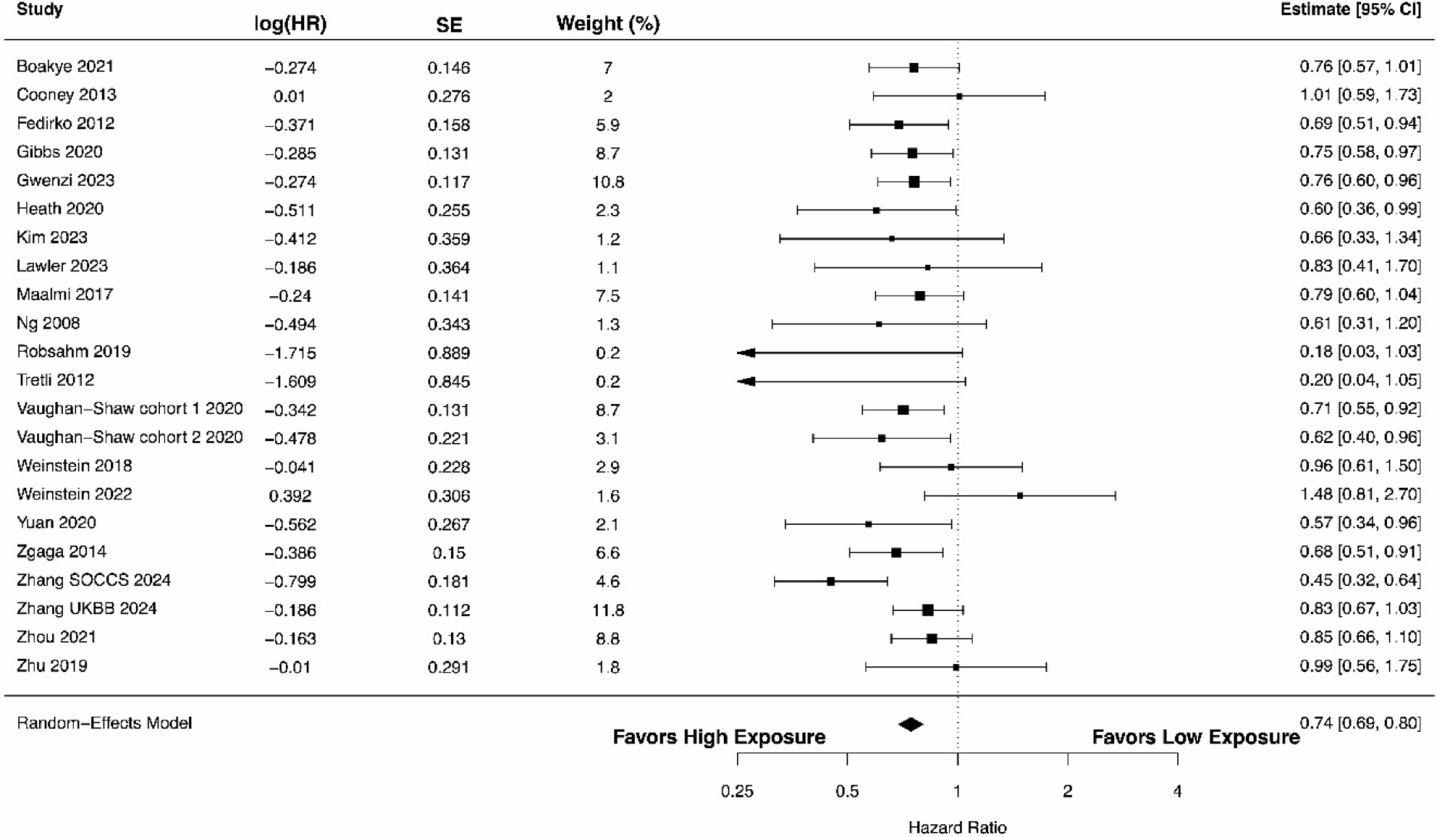
A forest plot showing the association between high versus low circulating 25(OH)D levels and colorectal cancer-specific survival

We conducted a pooled analysis to evaluate the association between circulating 25(OH)D levels (high vs. low) and CRC-specific survival. The forest plot shown in Fig. 2 summarizes 22 estimates from 20 studies [6, 34–36, 40, 48, 53, 56, 60, 62–64, 66, 67, 71, 74, 77–79, 81], presenting HRs with 95% CIs for individual studies and the overall effect. The pooled HR was 0.74 (95% CI 0.69–0.80), indicating significantly improved survival associated with higher 25(OH)D levels (*P* < 0.00001). Heterogeneity among studies was low, with an I^2^ of 0.01% (Cochran’s Q = 27.03). The estimated between-study variance was negligible with τ^2^ of 0.000003. The 95% prediction interval ranged from 0.69 to 0.80, indicating a consistent association across studies.

**Fig. 3:**
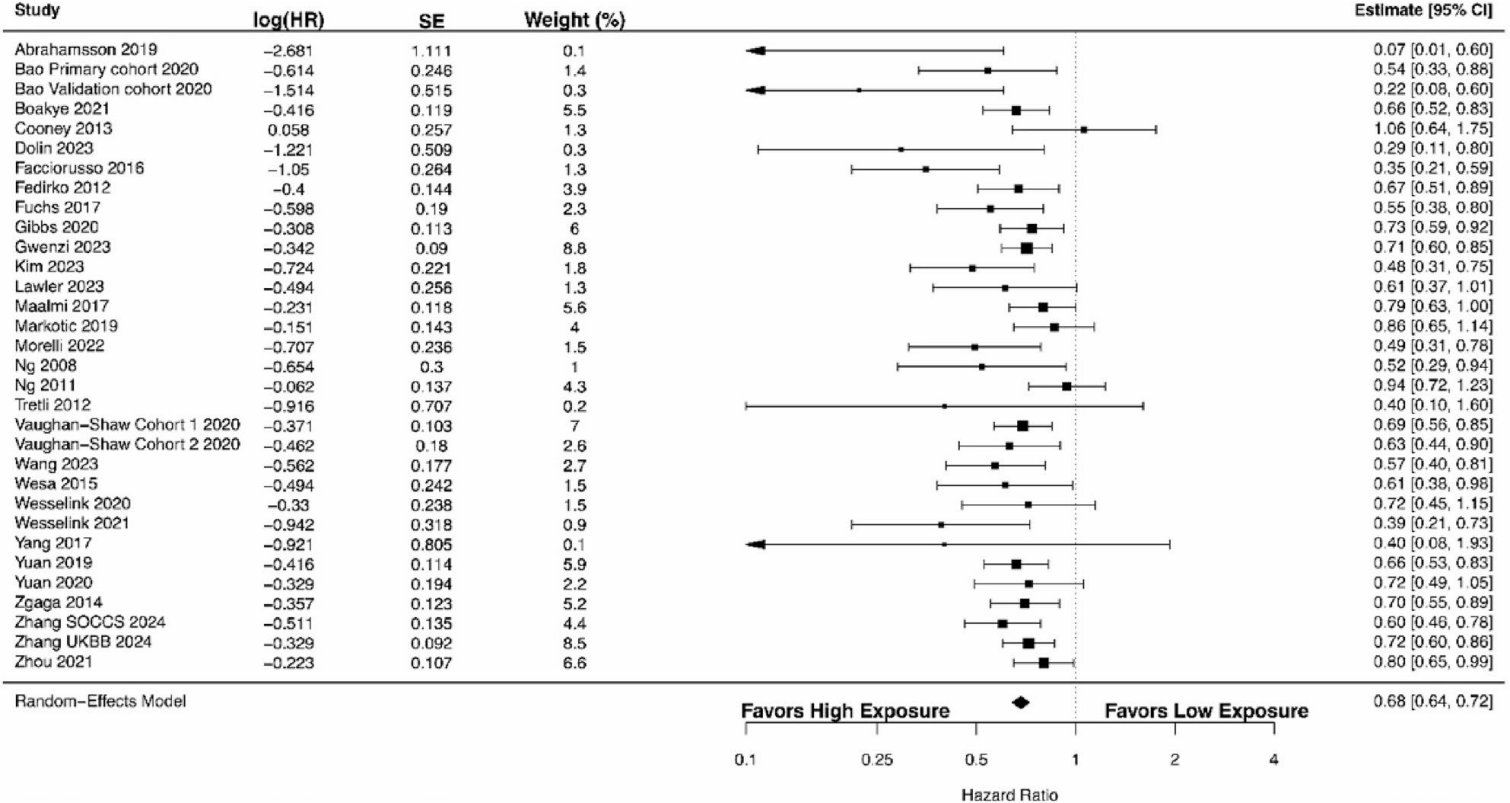
A forest plot showing the association between high versus low circulating 25(OH)D levels and overall survival in colorectal cancer patients

Random-effects models using data from 32 estimates across 29 studies [6, 33–36, 40, 43, 44, 47–49, 55, 58, 61, 62, 64–67, 70, 71, 73, 76–79, 81, 82, 86] revealed an overall HR of 0.68 (95% CI 0.64–0.72). As shown in Fig. 3, there was a significant association between higher 25(OH)D levels and improved OS (*P* < 0.00001). Between-study heterogeneity was low as indicated by an I^2^ value of 7.6% (Cochran’s Q = 48.39; τ² = 0.0021). The 95% prediction interval ranged from 0.61 to 0.76. In the subgroup analysis of metastatic CRC patients across 5 studies [33, 43, 44, 58, 73] (Supplementary Figure S1), the pooled HR for OS was 0.61 (95% CI 0.44–0.83; *P* = 0.002; Cochran’s Q = 14.07; I^2^ = 74%; τ² = 0.0903). However, the 95% prediction interval ranged from 0.31 to 1.18, indicating that although the overall association was statistically significant, the effect may vary across settings or populations. In the subgroup analysis of stage III CRC patients across 3 studies with 4 estimates [47, 61, 86] (Supplementary Figure S2), the pooled HR for OS was 0.54 (95% CI 0.43–0.67; *P* < 0.00001; Cochran’s Q = 3.16; I² = 0%; τ² = 0.0000007). The 95% prediction interval ranged from 0.43 to 0.67, suggesting that although the pooled association was statistically significant, the magnitude of effect may vary across different clinical settings.

To assess the robustness of our findings and the impact of differences in 25(OH)D categorization across studies, sensitivity analyses were conducted. Restricting the analysis to studies with comparable vitamin D thresholds (≥ 50 nmol/L or ≥ 20 ng/mL vs. <30 nmol/L or < 12 ng/mL) showed consistent results for CRC-specific survival (5 studies [48, 62, 77–79]; HR 0.76; 95% CI 0.66–0.88; *P* = 0.0001; Cochran’s Q = 0.27; I² = 0%; τ² = 0.00000), as presented in Supplementary Figure S3. Similarly, the association remained unchanged in the sensitivity analysis for OS (5 studies [48, 62, 77–79]; HR 0.71; 95% CI 0.64–0.80; *P* < 0.0001; Cochran’s Q = 4.32; I² = 0%; τ² = 0.00000646) (Supplementary Figure S4). Excluding studies rated as low quality did not substantially change the pooled estimates for CRC-specific survival (19 studies with 21 estimates [6, 34–36, 40, 48, 53, 56, 60, 62–64, 66, 67, 71, 77–79, 81]; HR 0.74; 95% CI 0.68–0.80; *P* < 0.0001; Cochran’s Q = 21.92; I² = 0%; τ² = 0.00000449) or for OS (28 studies with 31 estimates [6, 33–36, 40, 43, 44, 47–49, 55, 58, 61, 62, 64–67, 70, 71, 76–79, 81, 82, 86]; HR 0.69; 95% CI 0.65–0.73; *P* = 0.0001; Cochran’s Q = 46.43; I² = 1%; τ² = 0.00027), as shown in Supplementary Figures S5-S6, supporting the robustness of the main findings.

In the subgroup analyses stratified by timing of 25(OH)D measurement, the association between higher circulating 25(OH)D levels and improved survival outcomes remained consistent. For CRC-specific survival, the pooled HR was 0.81 (95% CI 0.70–0.94, I² = 0%) in pre-diagnostic studies [6, 34, 53, 60, 66, 71, 74, 78, 79] (Supplementary Figure S7) and 0.73 (95% CI 0.66–0.82; I² = 0%) in post-diagnostic studies [35, 36, 40, 48, 56, 64, 67, 77] (Supplementary Figure S8). Similarly, for OS, the pooled HR was 0.65 (95% CI 0.56–0.76; I² = 25.4%) in pre-diagnostic studies [6, 34, 47, 66, 71, 78, 79] (Supplementary Figure S9) and 0.67 (95% CI 0.61–0.73; I² = 32.9%) in post-diagnostic studies [33, 35, 36, 40, 43, 44, 48, 49, 55, 58, 61, 64, 65, 67, 70, 73, 76, 77, 82, 86] (Supplementary Figure S10).

### Association of circulating 25(OH)D levels with recurrence-free survival, time to CRC recurrence and disease-free survival

**Fig. 4:**
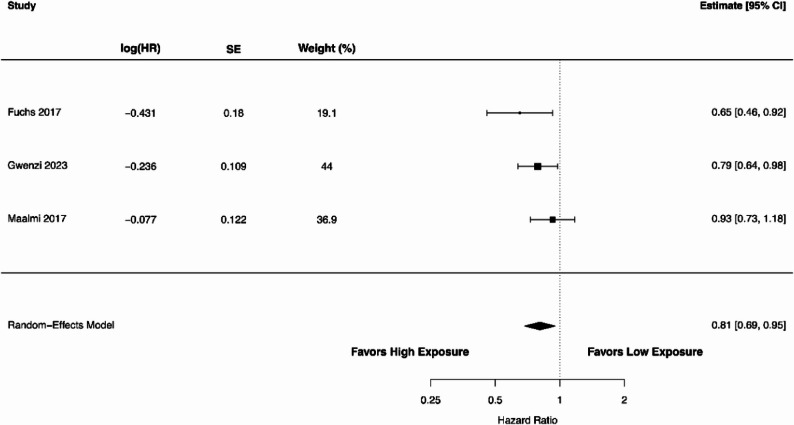
A forest plot showing the association between high versus low circulating 25(OH)D levels and recurrence-free survival in colorectal cancer patients

**Fig. 5:**
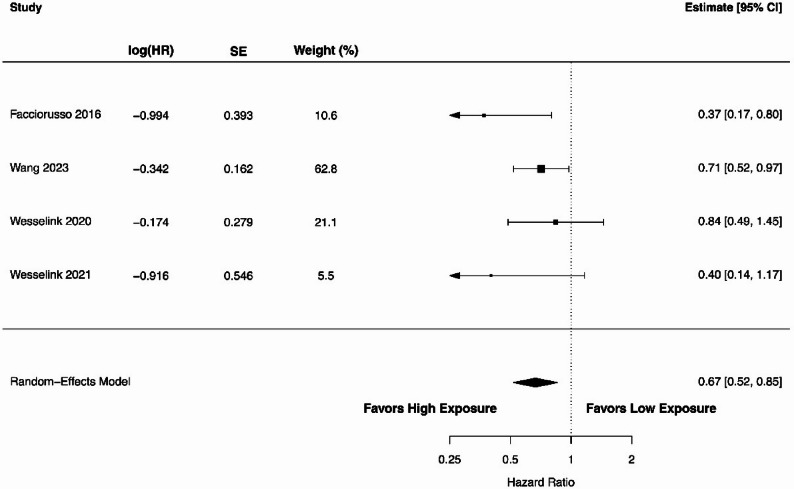
A forest plot showing the association between high versus low circulating 25(OH)D levels and time to recurrence in colorectal cancer patients

**Fig. 6:**
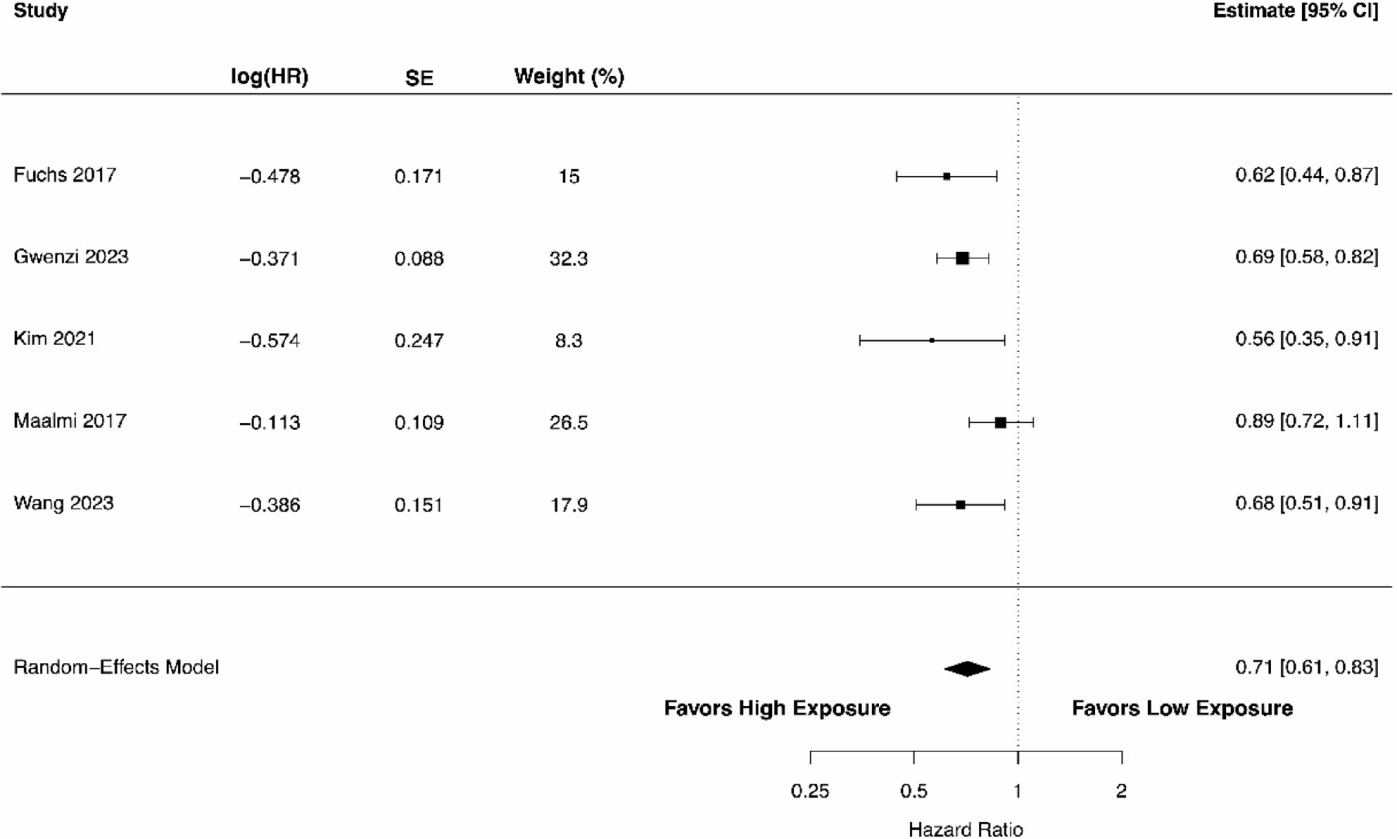
A forest plot showing the association between high versus low circulating 25(OH)D levels and disease-free survival in colorectal cancer patients

We analysed the relationship between circulating 25(OH)D levels (high vs. low) and RFS using data from three studies [47, 48, 77]. The forest plot in Fig. 4 demonstrates an HR of 0.81 (95% CI 0.69–0.95), indicating a statistically significant association between higher 25(OH)D levels and prolonged RFS (*P* = 0.010). The heterogeneity among the included studies was 18% (Cochran’s Q = 2.76), and the estimated between-study variance was small (τ² = 0.0038). The 95% prediction interval ranged from 0.66 to 0.99. The pooled analysis of four studies [44, 65, 70, 86] showed a significant association of higher 25(OH)D levels with longer TTR as shown in Fig. 5. The overall HR was 0.67 (95% CI 0.52–0.85; *P* = 0.001). The heterogeneity among studies was negligible, with an I² of 0% (Cochran’s Q = 3.96; τ² = 0.000003). The 95% prediction interval ranged from 0.52 to 0.85 for TTR. As shown in Fig. 6, the association of 25(OH)D levels with DFS was also significant with the pooled HR of 0.71 (95% CI 0.61–0.83; *P* < 0.00001) in five studies [47, 48, 77, 85, 86]. There was a substantial heterogeneity with an I^2^ value of 36.8% (Cochran’s Q = 6.02; τ² = 0.0106). The 95% prediction interval ranged from 0.55 to 0.92, indicating that the association remained consistent across studies.

**Fig. 7:**
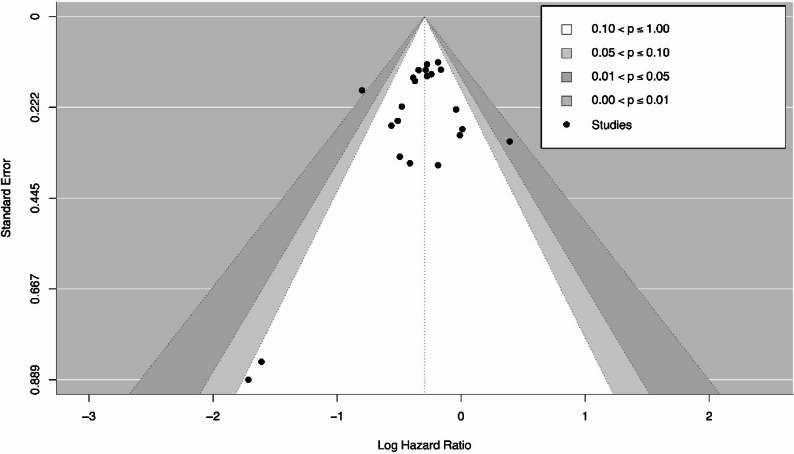
A funnel plot assessing potential publication bias in the meta-analysis of the association between circulating 25(OH)D levels and colorectal cancer-specific survival

### Analysis of publication bias

The median risk of bias score was 7 (interquartile range 6–8). There were 28 studies with high quality, 26 studies with fair quality, and 7 studies having high risk of bias. A frequent source of bias was the lack of representativeness of the exposed cohort, as many studies relied on selective populations, limiting generalizability. This bias may affect the pooled estimates in the systematic review by introducing heterogeneity, reducing precision, or inflating the observed associations. Publication bias was evaluated by visual inspection of a contour-enhanced funnel plot of the association between circulating 25(OH)D levels and CRC survival because more than 20 studies were available for this primary outcome (Fig. 7). The plot demonstrated studies distributed relatively symmetrically around the pooled effect estimate. Egger’s regression did not indicate significant small-study effects (z = − 1.29; *P* = 0.196). The trim-and-fill analysis imputed two potentially missing studies; however, after adjustment, the pooled effect estimate was minimally changed from HR 0.74 (95% CI 0.69–0.80) to HR 0.75 (95% CI 0.69–0.81), remaining highly statistically significant (*P* < 0.0001). Overall, these findings suggest that small-study effects are unlikely to have significantly influenced the results.

### Certainty of evidence

According to the GRADE assessment, the certainty of evidence was rated as low for CRC-specific survival. The certainty was rated as very low for OS. As the evidence was derived from observational studies, the initial certainty was rated as low and was further downgraded due to concerns regarding publication bias, while no serious limitations were identified in the domains of risk of bias, inconsistency, indirectness, or imprecision. For DFS, TTR, and RFS, the certainty was rated as very low due to imprecision related to the limited number of studies and additional concerns regarding publication bias. Detailed GRADE assessments are provided in Supplementary Table S4.

### Ongoing clinical trials evaluating vitamin D or vitamin D receptor polymorphisms

To contextualize our findings in terms of biomarker and clinical relevance, we summarized ongoing active clinical trials investigating the prognostic and therapeutic roles of circulating 25(OH)D levels or VDR polymorphisms in the CRC population (Supplementary Table S7). These studies primarily focus on vitamin D supplementation, the integration of vitamin D with chemotherapy regimens, and its potential effects on modulating clinical outcomes. Remarkably, while trials are investigating circulating 25(OH)D as a modifiable biomarker, only one currently incorporates genetic analysis of VDR.

## Discussion

This systematic review and meta-analysis highlights the relationship between circulating 25(OH)D levels and CRC outcomes, as well as the potential prognostic implications of VDR gene polymorphisms. CRC patients across all stages from diverse geographical and ethnic backgrounds were included, reflecting substantial variability in study populations and follow-up durations.

Our findings indicate that higher circulating 25(OH)D levels were significantly associated with better prognostic outcomes, including improved CRC-specific survival and OS. CRC patients with higher or sufficient circulating 25(OH)D levels had a 26% lower risk of death from CRC and a 32% lower risk of overall death. In addition, higher 25(OH)D levels in CRC patients were associated with a 19% lower risk of recurrence or death as measured by RFS, a 39% longer TTR, and a 26% lower risk of experiencing disease or death. Our results add to the growing body of evidence on the association between circulating 25(OH)D levels and CRC outcomes. Compared to prior meta-analyses, this study includes a larger dataset with 20 studies for CRC-specific survival and 29 studies for OS. For instance, Maalmi et al. [87] across 5 studies and Maalmi et al. [88] across 11 studies reported comparable HRs for CRC-specific survival (0.65 and 0.67, respectively) and OS (0.71 and 0.68, respectively). Similarly, compared to Wu et al. [89] and Li et al. [90], our findings align closely but expand upon previous work by including additional studies. Likewise, our pooled estimates are consistent with a previous report including 11 studies that supported circulating 25(OH)D as a potential prognostic indicator in CRC [91]. Previously, one meta-analysis suggested an increased risk of mortality and progression in stage III CRC patients with low circulating 25(OH)D levels before surgery [7]. In contrast, a previous general population-based study observed no significant relationship between 25(OH)D and CRC mortality [92]. By synthesizing more recent evidence, our analysis provides comprehensive up-to-date insights into the potential prognostic role of vitamin D.

VDR SNPs may contribute to clinical outcomes in CRC, given their role in regulating genes associated with cell differentiation, inflammation, and apoptosis [93]. Existing meta-analyses have reported the impact of VDR SNPs on the risk of CRC with varying findings [24, 94, 95]. A recent meta-analysis of gastrointestinal cancers, including colorectal, gastric, and esophageal malignancies, demonstrated significant associations between VDR SNPs and increased cancer risk, particularly with the *BsmI* polymorphism [15]. Similarly, another meta-analysis reported that high circulating vitamin D levels were significantly associated with a lower cancer risk [96]. This effect may be modified by specific VDR genotypes, with an inverse association observed mainly in carriers of the *TaqI* Tt/tt variants. Nonetheless, to date, the evidence is not sufficient to ascertain the role of VDR polymorphisms in prognosis, particularly survival outcomes in the CRC population. In contrast to the quantitative findings for circulating 25(OH)D in the present study, the available evidence on VDR polymorphisms remains limited and exploratory.

In our systematic review, the variability in reported associations between VDR polymorphisms and CRC survival outcomes across studies highlights the challenges in drawing definitive conclusions. As described, varying results have been observed for *ApaI*, *BsmI*, *FokI*, *TaqI*, and *Cdx2* polymorphisms, with some studies reporting significant associations with survival outcomes while others reporting no association. For example, *BsmI* wild-type alleles were associated with worse OS in one study [80], whereas no association was observed in others [16, 50]. Similarly, while Messaritakis et al. [16] identified *FokI* homozygous mutant genotypes as potential predictors of lower survival, other studies found no association [37, 50]. These discrepancies might arise from differences in population genetics, environmental factors, and statistical adjustments for confounders. In our study, alleles and genotypes were not directly comparable across studies due to variations in genetic background and methodology, precluding a robust meta-analysis. Thus, the findings related to VDR polymorphisms should be interpreted with caution and considered hypothesis-generating rather than confirmatory. While data heterogeneity limits quantitative synthesis of VDR polymorphisms, the narrative synthesis highlights potential genotype-specific therapeutic considerations in this population. However, these possibilities remain preliminary and require further validation. The current findings predominantly reflect data from studies conducted in European populations, which may limit the applicability of these results to other ethnic groups. VDR polymorphism frequencies are known to vary across populations, with significant differences reported among European, Asian, and African cohorts [97, 98]. Future research should prioritize investigating VDR polymorphisms and CRC prognostic outcomes in more diverse populations to better understand the role of genetic and environmental interactions in CRC prognosis. Larger prospective studies with standardized methodologies that account for potential confounders and interactions are warranted [99].

Our study has several strengths. It provides a comprehensive analysis of circulating 25(OH)D levels and VDR polymorphisms in CRC prognosis, with a large sample size, ensuring robust statistical power. The inclusion of multiple survival outcomes (CRC-specific survival, OS, and RFS) provides an informative understanding of vitamin D’s potential prognostic role. The moderate heterogeneity observed among outcomes underscores the importance of rigorous study quality assessment, which enhances the reliability of our pooled estimates. However, there are some limitations. First, the observational nature of the included studies limits causal inference, as potential residual confounders might not have been fully ruled out. It is difficult to conclude that survival outcomes are causally related to 25(OH)D concentrations. In particular, the possibility of reverse causality should be considered, as patients with more advanced or aggressive CRC may experience poorer nutritional status, systemic inflammation, and cancer-related cachexia, leading to lower circulating 25(OH)D levels. Moreover, systemic inflammation may itself suppress circulating 25(OH)D concentrations, potentially contributing to inflammation-induced depression of vitamin D levels independent of nutritional status. Although many studies adjusted for major prognostic factors such as age, sex, and tumor stage, adjustment for other confounders, such as body mass index, systemic inflammatory markers, and detailed treatment characteristics, was less consistent. Second, substantial variability in 25(OH)D measurement methods and study designs may introduce bias and limit stratified analyses. This includes differences in assay techniques (e.g., radioimmunoassay vs. mass spectrometry-based assay), the timing of blood collection, potential seasonal variation, and follow-up durations. Furthermore, heterogeneous definitions of “high” and “low” 25(OH)D levels, including study-specific quantiles, and variation in baseline patient characteristics across the included cohorts may contribute to between-study heterogeneity. Although subgroup analyses were conducted based on pre-diagnostic and post-diagnostic measurements, the post-diagnostic category may still encompass heterogeneous clinical contexts, including peri-operative and during treatment periods, which may reflect distinct biological mechanisms and treatment-related influences on 25(OH)D levels. The inability to further stratify these time points due to inconsistent reporting across studies may limit the interpretability of the findings. Although the highest-versus-lowest comparison was used to harmonize exposure across studies, heterogeneity due to differences in vitamin D cut-off values and categorization methods (quartiles, tertiles, or predefined clinical thresholds) may limit the comparability of pooled estimates and should be considered when interpreting the findings. By restricting analyses to studies with harmonized clinical thresholds in sensitivity analyses, the comparability of exposure definitions was improved, partially addressing this limitation; however, residual heterogeneity cannot be fully excluded. Moreover, further stratified analyses by treatment modality were not feasible because most studies included mixed-treatment populations and did not report treatment-specific HRs. Third, inconsistent reporting of VDR polymorphism analyses, particularly regarding haplotypes, as well as differences in the genetic models applied (dominant or recessive) may limit the interpretation and comparability of genetic findings. Moreover, inconsistencies in the selection of covariates for adjustment and the limited evaluation of potential gene-environment interactions may further challenge the interpretation. Additionally, a formal dose-response meta-analysis was not performed because circulating 25(OH)D levels were categorized heterogeneously across studies, with varying cut-off values and inconsistent reference categories, thereby precluding appropriate modeling of a dose-response relationship. Furthermore, although a structured and systematic search strategy was applied, EMBASE and Web of Science were not included due to access limitations in this systematic review. Therefore, relevant studies indexed exclusively in these databases may have been missed, and the search may not be considered fully comprehensive. Lastly, most studies were conducted in European and North American populations, with limited representation from Asia and Africa, which may limit the generalizability of our results. Heterogeneity in genetic profiles across populations may further influence both vitamin D metabolism and VDR polymorphism distributions, potentially affecting the observed associations. The underrepresentation of non-European populations in the included studies restricts the ability to assess the consistency of observed associations across diverse ethnic groups. Future large-scale studies should address these gaps by including more diverse cohorts.

The positive association between circulating 25(OH)D and CRC survival outcomes observed in our study may suggest potential prognostic relevance as an observational biomarker, primarily for hypothesis generation at this stage. While the findings suggest a possible role for vitamin D status in prognostic assessment, more evidence is necessary to support its clinical utility. While regular monitoring of 25(OH)D levels in this population may offer additional context for prognostic evaluation, this interpretation remains speculative and should be interpreted with caution. Further prospective interventional studies are warranted before drawing practice-guiding implications. The heterogeneous findings regarding VDR polymorphisms underscore the need for future comprehensive genomic studies. The combination of genomic data with clinical and biochemical parameters may help refine prognostic stratification in future research settings. Understanding the relationship between circulating 25(OH)D and VDR genetic variants may offer biological and prognostic insights, especially in populations at high risk of CRC.

## Conclusion

This systematic review and meta-analysis highlights that higher circulating 25(OH)D levels are associated with better survival outcomes. While the prognostic impact of VDR polymorphisms remains inconclusive, current evidence suggests that certain VDR variants may be associated with variation in CRC outcomes. From epidemiologic and biological perspectives, integrated multidimensional approaches that combine serum 25(OH)D assessment with clinical genotyping are needed to better characterize their potential prognostic relevance. Prospective studies combining both biochemical and genomic biomarkers are warranted to clarify their role in risk and outcome assessment. Most importantly, a deeper understanding of the interplay between vitamin D status, VDR genetic variants, and CRC progression may provide biological insights into disease mechanisms. At present, these observations should be considered hypothesis-generating rather than indicative of direct practice-oriented implications. Further validation in large, well-designed prospective and interventional studies is required before routine clinical applications, including monitoring for prognostic purposes, can be considered.

## Supplementary Information

Supplementary Material 1.

Supplementary Material 2.

Supplementary Material 3.

Supplementary Material 4.

Supplementary Material 5.

Supplementary Material 6.

Supplementary Material 7.

Supplementary Material 8.

## Data Availability

The datasets used and/or analysed during the current study are available from the corresponding author on reasonable request.

## Abbreviations

25(OH)D: 25-hydroxyvitamin D
CI: Confidence interval
CRC: Colorectal cancer
EMT: Epithelial-mesenchymal transition
GRADE: Grading of Recommendations Assessment, Development, and Evaluation
HR: Hazard ratio
HWE: Hardy-Weinberg equilibrium
LEF-1: Lymphoid enhancer factor
PRISMA: Preferred Reporting Items for Systematic Reviews and Meta-Analyses
SNP: Single nucleotide polymorphism
TME: Tumor microenvironment
VDR: Vitamin D receptor
