# Supplementary material for: Circulating vitamin D status and prognosis in colorectal cancer: a systematic review and meta-analysis with exploratory evidence on vitamin D receptor polymorphisms
Source: BMC Cancer. 2026 Apr 16;26:687. doi: 10.1186/s12885-026-16026-x (PMC13220566; doi:10.1186/s12885-026-16026-x)
Supplement: Supplementary file 2 — Supplementary Material 2. [file 12885_2026_16026_MOESM2_ESM.docx]

**Supplementary Table S2** Search strategies

| **Database** | **Search terms** |
| --- | --- |
| CochraneLibrary | ("vitamin d receptor gene polymorphism" OR "VDR gene polymorphism" OR VDR OR "vitamin D receptor" OR "vitamin d receptor polymorphism" OR "VDR polymorphism" OR "VDR gene" OR "vitamin d receptor gene" OR ApaI OR BsmI OR FokI OR TaqI OR rs7975232 OR rs1544410 OR rs2228570 OR rs731236 OR "25-hydroxyvitamin D" OR "serum 25-hydroxyvitamin D" OR calcidiol OR 25OHD OR "serum vitamin D" OR "circulating vitamin D"):ti,ab,kw AND ("colorectal cancer"):ti,ab,kw |
| ScienceDirect | ((“vitamin d receptor gene polymorphism” OR “VDR gene polymorphism” OR “vitamin d receptor polymorphism” OR “vitamin D receptor” OR “25-hydroxyvitamin D” OR calcidiol OR “serum vitamin D” OR 25OHD) AND (“colorectal cancer”)) |
| Scopus | (TITLE-ABS-KEY(“vitamin d receptor gene polymorphism”) OR TITLE-ABS-KEY(“VDR polymorphism”) OR TITLE-ABS-KEY(“VDR ApaI”) OR TITLE-ABS-KEY(ApaI) OR TITLE-ABS-KEY(“Apa I polymorphism”) OR TITLE-ABS-KEY(“VDR BsmI”) OR TITLE-ABS-KEY(BsmI) OR TITLE-ABS-KEY(“BsmI polymorphism”) OR TITLE-ABS-KEY(“VDR FokI”) OR TITLE-ABS-KEY(FokI) OR TITLE-ABS-KEY(“FokI polymorphism”) OR TITLE-ABS-KEY(“VDR TaqI”) OR TITLE-ABS-KEY(TaqI) OR TITLE-ABS-KEY(“TaqI polymorphism”) OR TITLE-ABS-KEY(“VDR gene polymorphism”) OR TITLE-ABS-KEY(“25-hydroxyvitamin D”) OR TITLE-ABS-KEY(“serum vitamin D”) OR TITLE-ABS-KEY(calcidiol) OR TITLE-ABS-KEY(“circulating vitamin D”) OR TITLE-ABS-KEY(25OHD) OR TITLE-ABS-KEY(“serum 25-hydroxyvitamin D”) OR TITLE-ABS-KEY(rs7975232) OR TITLE-ABS-KEY(rs1544410) OR TITLE-ABS-KEY(rs2228570) OR TITLE-ABS-KEY(rs731236)) AND TITLE-ABS-KEY(“colorectal cancer”) |
| PubMed | **((((((((((((((((((((((((((((((vitamin d receptor[MeSH Terms]) OR ("vitamin d receptor gene polymorphism"[Title/Abstract])) OR ("VDR gene polymorphism"[Title/Abstract])) OR ("vitamin d receptor polymorphism"[Title/Abstract])) OR ("VDR polymorphism"[Title/Abstract])) OR (VDR[Title/Abstract])) OR ("vitamin d receptor"[Title/Abstract])) OR ("VDR gene"[Title/Abstract])) OR ("vitamin d receptor gene"[Title/Abstract])) OR (ApaI[Title/Abstract])) OR (BsmI[Title/Abstract])) OR (FokI[Title/Abstract])) OR (TaqI[Title/Abstract])) OR ("ApaI polymorphism"[Title/Abstract])) OR ("BsmI polymorphism"[Title/Abstract])) OR ("FokI polymorphism"[Title/Abstract])) OR ("TaqI polymorphism"[Title/Abstract])) OR (rs7975232[Title/Abstract])) OR (rs1544410[Title/Abstract])) OR (rs2228570[Title/Abstract])) OR (rs731236[Title/Abstract])) OR ("25-hydroxyvitamin D"[Title/Abstract])) OR (calcidiol[MeSH Terms])) OR (calcidiol[Title/Abstract])) OR (25OHD[Title/Abstract])) OR ("serum vitamin D"[Title/Abstract])) OR ("circulating vitamin D"[Title/Abstract])) OR ("serum 25-hydroxyvitamin D"[Title/Abstract])) AND (((colorectal neoplasm[MeSH Terms]) OR (cancer, colorectal[MeSH Terms])) OR ("colorectal cancer"[Title/Abstract]))) AND (human[MeSH Terms])) AND (english[Language])** |
