# Supplementary material for: Circulating vitamin D status and prognosis in colorectal cancer: a systematic review and meta-analysis with exploratory evidence on vitamin D receptor polymorphisms
Source: BMC Cancer. 2026 Apr 16;26:687. doi: 10.1186/s12885-026-16026-x (PMC13220566; doi:10.1186/s12885-026-16026-x)
Supplement: Supplementary file 3 — Supplementary Material 3. [file 12885_2026_16026_MOESM3_ESM.docx]

**Supplementary Table S3** Quality assessment

| **Cohort studies** | | | | | | | | | | | | | | | | | |
| --- | --- | --- | --- | --- | --- | --- | --- | --- | --- | --- | --- | --- | --- | --- | --- | --- | --- |
| **Author, year** | **Representativeness of the exposed cohort** | | | **Selection of the non-exposed cohort** | | **Ascertainment of exposure** | | **Outcome not present at the beginning** | **Comparability of cohorts** | | **Assessment of the outcome** | | **Follow-up duration** | | **Adequacy of follow-up of cohorts** | | **Quality of the study** |
| Abrahamsson et al., 2019 | * | | | * | | * | | * | ** | | * | | * | | * | | 9 |
| Abrahamsson et al., 2021 | * | | | * | | * | | * | ** | | * | | * | | * | | 9 |
| Bao et al., 2020 | * | | | * | | * | | * | * | | * | | * | | * | | 8 |
| Berger et al., 2018 | * | | | * | | * | | * | * | | * | | * | | - | | 7 |
| Boakye et al., 2021 | - | | | * | | * | | * | ** | | * | | * | | * | | 8 |
| Cooney et al., 2013 | - | | | * | | * | | * | ** | | * | | * | | - | | 7 |
| Cuomo, 2024 | * | | | * | | * | | * | ** | | * | | * | | * | | 9 |
| De Mattia et al., 2018 | - | | | * | | * | | * | ** | | * | | - | | - | | 6 |
| De Mattia et al., 2019 | - | | | * | | * | | * | ** | | * | | * | | - | | 7 |
| De Mattia et al., 2021 | - | | | * | | * | | * | ** | | * | | * | | - | | 7 |
| Dolin et al., 2023 | * | | | * | | * | | * | * | | * | | * | | * | | 8 |
| Egan et al., 2010 | - | | | * | | * | | * | * | | * | | - | | - | | 5 |
| Facciorusso et al., 2016 | * | | | * | | * | | * | * | | * | | * | | * | | 8 |
| Fedirko et al., 2012 | - | | | * | | * | | * | ** | | * | | * | | - | | 7 |
| Fuchs et al., 2017 | * | | | * | | * | | * | ** | | * | | * | | * | | 9 |
| Gibbs et al., 2020 | * | | | * | | * | | * | ** | | * | | * | | * | | 9 |
| Giessen et al., 2014 | - | | | * | | * | | * | * | | - | | * | | * | | 6 |
| Giessen-Jung et al., 2015 | - | | | * | | * | | * | * | | - | | * | | * | | 6 |
| Gwenzi et al., 2023 | - | | | * | | * | | * | ** | | * | | * | | * | | 8 |
| Hamada et al., 2018 | - | | | * | | * | | * | * | | * | | * | | * | | 7 |
| Heath et al., 2020 | - | | | * | | * | | * | ** | | * | | * | | - | | 7 |
| Jacobs et al., 2007 | - | | | * | | * | | * | * | | - | | - | | - | | 4 |
| Kim et al., 2021 | - | | | * | | * | | * | ** | | * | | * | | * | | 8 |
| Kim et al., 2023 | - | | | * | | * | | * | ** | | * | | * | | - | | 7 |
| Lawler et al., 2023 | * | | | * | | * | | * | ** | | * | | - | | - | | 7 |
| Maalmi et al., 2017 | * | | | * | | * | | * | ** | | * | | * | | * | | 9 |
| Markotic et al., 2019 | * | | | * | | * | | * | ** | | * | | * | | * | | 9 |
| Messaritakis et al., 2020 | - | | | * | | * | | * | * | | * | | * | | - | | 6 |
| Messaritakis et al., 2022 | * | | | * | | * | | * | - | | - | | - | | - | | 4 |
| Messaritakis et al., 2023 | * | | | * | | * | | * | * | | * | | - | | - | | 6 |
| Mezawa et al., 2010 | * | | | * | | * | | * | ** | | * | | * | | - | | 8 |
| Morelli et al., 2022 | * | | | * | | * | | * | - | | - | | - | | - | | 4 |
| Ng et al., 2008 | - | | | * | | * | | * | ** | | * | | * | | * | | 8 |
| Ng et al., 2011 | * | | | * | | * | | * | ** | | * | | * | | - | | 8 |
| Obermannova et al., 2015 | - | | | * | | * | | * | * | | - | | * | | * | | 6 |
| Pérez-Durán et al., 2023 | - | | | * | | * | | * | ** | | * | | * | | * | | 8 |
| Perna et al., 2013 | - | | | * | | - | | * | * | | - | | * | | - | | 4 |
| Robsahm et al., 2019 | - | | | * | | * | | * | ** | | * | | - | | - | | 6 |
| Sinicrope et al., 2021 | * | | | * | | * | | * | * | | * | | - | | - | | 6 |
| Slattery et al., 2014 | - | | | * | | * | | * | ** | | * | | * | | - | | 7 |
| Torfadottir et al., 2019 | - | | | * | | * | | * | ** | | * | | * | | - | | 7 |
| Tretli et al., 2012 | - | | | * | | * | | * | * | | * | | - | | * | | 6 |
| Vaughan-Shaw et al., 2020 | * | | | * | | * | | * | * | | * | | * | | * | | 8 |
| Wang et al., 2023 | * | | | * | | * | | * | ** | | * | | * | | * | | 9 |
| Weinstein et al., 2018 | - | | | * | | * | | * | ** | | - | | * | | - | | 6 |
| Weinstein et al., 2022 | - | | | * | | - | | * | ** | | - | | * | | - | | 5 |
| Wesa et al., 2015 | - | | | * | | * | | * | * | | - | | * | | * | | 6 |
| Wesselink et al., 2020 | * | | | * | | * | | * | ** | | * | | * | | - | | 8 |
| Wesselink et al., 2021 | * | | | * | | * | | * | ** | | * | | * | | * | | 9 |
| Xia et al., 2022 | * | | | * | | * | | * | - | | * | | - | | - | | 5 |
| Yang et al., 2017 | * | | | * | | * | | * | * | | * | | * | | * | | 8 |
| Yuan et al., 2019 May | * | | | * | | * | | * | * | | * | | * | | * | | 8 |
| Yuan et al., 2019 | * | | | * | | * | | * | ** | | * | | * | | * | | 9 |
| Yuan et al., 2020 | - | | | * | | * | | * | ** | | * | | * | | * | | 8 |
| Zgaga et al., 2014 | - | | | * | | * | | * | * | | - | | * | | * | | 6 |
| Zhang et al., 2024 | * | | | * | | - | | * | ** | | * | | * | | * | | 8 |
| Zhou et al., 2021 | - | | | * | | * | | * | ** | | * | | * | | * | | 8 |
| Zhu et al., 2017 | * | | | * | | * | | * | * | | * | | * | | * | | 8 |
| Zhu et al., 2019 | - | | | * | | * | | * | * | | * | | * | | * | | 7 |
| **Case-control studies** | | | | | | | | | | | | | | | | | |
| **Author, year** | **Adequate case definition** | | | **Representativeness of the cases** | | **Selection of controls** | | **Definition of controls** | **Comparability of cases and controls** | | **Ascertainment of exposure** | | **Ascertainment of method** | | **Nonresponse rate** | | **Quality of the study** |
| Väyrynen et al., 2016 | - | | | * | | * | | * | * | | * | | * | | * | | 7 |
| **Cross-sectional studies** | | | | | | | | | | | | | | | | | |
| **Author, year** | | **Representativeness of the sample** | **Sample size** | | **Non-respondents** | | **Ascertainment of the exposure** | | | **Confounders are controlled** | | **Assessment of the outcome** | | **Statistical test** | | **Quality of the study** | |
| Jacobs et al., 2016 | | * | - | | - | | ** | | | * | | * | | * | | 6 | |
