# Supplementary material for: Circulating vitamin D status and prognosis in colorectal cancer: a systematic review and meta-analysis with exploratory evidence on vitamin D receptor polymorphisms
Source: BMC Cancer. 2026 Apr 16;26:687. doi: 10.1186/s12885-026-16026-x (PMC13220566; doi:10.1186/s12885-026-16026-x)
Supplement: Supplementary file 4 — Supplementary Material 4. [file 12885_2026_16026_MOESM4_ESM.docx]

**Supplementary Table S4** Summary of findings using the GRADE assessment

| **Outcomes** | **No. of studies** | **No. of Participants** | **Pooled HR (95% CI)** | **I^2^ (%)** | **Risk of bias** | **Imprecision** | **Inconsistency** | **Indirectness** | **Publication bias** | **Final certainty** |
| --- | --- | --- | --- | --- | --- | --- | --- | --- | --- | --- |
| CRC-specific survival | 20 | 31,421 | 0.74  (0.69–0.80) | 0.0 | Not serious | Not serious | Not serious | Not serious | Not serious | Low |
| Overall survival | 29 | 35,091 | 0.68  (0.64–0.72) | 7.6 | Not serious | Not serious | Not serious | Not serious | Serious | Very low |
| Recurrence-free survival | 3 | 6,745 | 0.81  (0.69–0.95) | 18.0 | Not serious | Serious | Not serious | Not serious | Serious | Very low |
| Time-to-recurrence | 4 | 3,428 | 0.67  (0.52–0.85) | 0.0 | Not serious | Serious | Not serious | Not serious | Serious | Very low |
| Disease-free survival | 5 | 8,977 | 0.71  (0.61–0.83) | 36.8 | Not serious | Not serious | Not serious | Not serious | Serious | Very low |
