# Supplementary material for: Circulating vitamin D status and prognosis in colorectal cancer: a systematic review and meta-analysis with exploratory evidence on vitamin D receptor polymorphisms
Source: BMC Cancer. 2026 Apr 16;26:687. doi: 10.1186/s12885-026-16026-x (PMC13220566; doi:10.1186/s12885-026-16026-x)
Supplement: Supplementary file 5 — Supplementary Material 5. [file 12885_2026_16026_MOESM5_ESM.docx]

**Supplementary Table S5** Characteristics of included studies

| **Author, year, country** | **CRC details (Sample size: Male/Female)** | **Treatments** | | | **Exposure measurements** | | **Follow- up duration** | **Prognostic outcomes** | | | | | **Other outcomes** | **Adjusted covariates** |
| --- | --- | --- | --- | --- | --- | --- | --- | --- | --- | --- | --- | --- | --- | --- |
|  |  | **Sx** | **RT** | **CMT** | **Vitamin D level** | **VDR SNP** |  | **OS** | **CSS** | **DFS** | **TTR/ Rec** | **RFS** |  |  |
| Abrahamsson et al., 2019, Norway [82] | Locally advanced rectal cancer (84: 50/34) | ✓ | ✓ | ✓ | Before Tx |  | 74.5 months*^b^* | ⊕ |  |  |  |  | ⊕ (PFS) | Age, sex, body mass index, season |
| Abrahamsson et al., 2021, Norway [83] | Locally advanced rectal cancer (129: 83/46) | ✓ | ✓ | ✓ | Before Tx |  | 39 months*^b^* |  | ⊕ |  |  |  |  | Disease stage, season |
| Bao et al., 2020, China [61] | Stage II – III CRC (523: 304/219) in primary cohort; Stage II – III CRC (205; 120/85) in validation cohort | ✓ |  | ✓ | Before Tx |  | 64.7 months*^b^* in primary cohort; 53 months^b^ in validation cohort | ⊕ |  |  |  |  |  | Age, sex, histology, T stage, N stage, adjuvant chemotherapy, no. of lymph nodes dissected |
| Berger et al., 2018, U.S. and Italy [18] | mCRC (295: 193/102) in discovery cohort; mCRC (227: 138/89) in validation cohort, | ✓ |  | ✓ |  | rs731236 | 65 months*^b^* in discovery cohort; 60 months*^b^* in validation cohort | ⊕ in discovery cohort; NS in validation cohort |  |  |  |  |  | Age, sex, ECOG performance status, primary tumor site, liver limited metastases, primary tumor resection, adjuvant chemotherapy, RAS status, and BRAF status |
| Boakye et al., 2021, Germany [67] | Stage I – IV CRC (2592: 1542/1050) | ✓ | ✓ | ✓ | Before Tx |  | 72 months*^b^* | ⊕ | NS |  |  |  |  | Age, sex, tumor stage, tumor site, year of diagnosis, years of school, education, body mass index, lifetime physical activity, smoking status, lifetime alcohol consumption, vegetable and fruit intake, time of blood draw with respect to season, chemo (radio) therapy initiation, CRC surgery, CCI score, tumor site × log(time), and body mass index × log(time), total thiol level |
| Cooney et al., 2013, U.S. [36] | Stage I – IV CRC (368: 216/152) | ✓ | ✓ | ✓ | After Dx |  | 64.8 years*^a^* | NS | NS |  |  |  |  | Age, sex, tumor stage, race/ ethnicity, smoking status, month of blood draw, C-reactive protein |
| Cuomo, 2024, U.S. [84] | Stage I - IV colon cancer (1602) |  |  |  | After Dx |  | 5 years | ⊕ |  |  |  |  |  | Cancer stage, gender, race/ ethnicity, tobacco dependence, obesity |
| De Mattia et al., 2018, Canada and Italy [51] | mCRC (247: 160/87) in discovery cohort; mCRC (167: 110/57) in replication cohort | ✓ |  | ✓ |  | rs1168287, rs2853564, rs4760648, rs11574077, rs12717991, rs11574026 |  |  |  |  |  |  | ⊖ GI toxicity, ⊖ Neutropenia in discovery cohort; NS in replication cohort | Age, sex, cancer site, stage at diagnosis, radical surgery, adjuvant chemotherapy |
| De Mattia et al., 2019, Canada and Italy [54] | mCRC (247: 160/87) in discovery cohort; mCRC (90: 60/30) in replication cohort | ✓ |  | ✓ |  | rs4760648, rs7299460 |  | ⊕ |  |  |  |  | NS (PFS) | Age, sex, cancer site, stage at diagnosis, radical surgery, adjuvant chemotherapy |
| De Mattia et al., 2021, Canada and Italy [68] | mCRC (243: 158/85) in discovery cohort; mCRC (92: 61/31) in replication cohort | ✓ |  | ✓ |  | rs7299460 |  | ⊖ |  |  |  |  |  | Age, sex, cancer site, radical surgery |
| Dolin et al., 2023, Denmark [76] | Stage I – III CRC (398: 221/177) | ✓ |  |  | Before Tx |  | 73 months*^b^* | ⊕ |  |  |  |  |  | Age, sex |
| Egan et al., 2010, U.S. [31] | Patients with resected colorectal adenoma (1439: 963/476) |  |  |  |  | 42 VDR SNPs including rs1544410, rs731236, rs11568820 |  |  |  |  |  |  | NS (Metachronous neoplasia) | Age, sex |
| Facciorusso et al., 2016, Italy [44] | mCRC (143: 102/41) |  | ✓ | ✓ | After Dx |  | 72 months*^b^* | ⊕ |  |  | ⊕ |  |  | Carcinoembryonic antigen, number of nodules, max diameter |
| Fedirko et al., 2012, Multicenters (Western countries) [34] | Stage I – IV CRC (1202: 596/606) |  |  |  | Before Dx | rs1544410, rs2228570 | 73 months*^a^* | ⊕ 25 (OH) D;  NS (VDR SNP) | ⊕ 25 (OH) D;  NS (VDR SNP) |  |  |  |  | Age, sex, cancer stage, tumor grade differentiation, primary tumour site, smoking status, body mass index, physical activity, season of blood collection, year of diagnosis, country of residence |
| Fuchs et al., 2017, U.S. [47] | Stage III CRC (1016: 572/444) |  |  | ✓ | Before Dx |  | 7.3 years*^b^* | ⊕ |  | ⊕ |  | ⊕ |  | Age, sex, family history of CRC, baseline performance status, depth of invasion through bowel wall, no. of positive lymph nodes, grade of tumor differentiation, treatment arm |
| Gibbs et al., 2020, Multicenters (Western countries) [62] | Stage I – IV CRC (1043) in EPIC cohort; Stage I – IV CRC (238) in CPS-II cohort |  |  |  | ✓ |  | 8.3 years*^a^* in EPIC cohort; 7.3 years*^a^* in CPS-II cohort | ⊕ | ⊕ |  |  |  |  | Year of diagnosis, sex, tumor site, body mass index, physical activity, smoking status, tumor stage, country |
| Giessen et al., 2014, Germany [38] | Stage I – III rectal cancer (256: 164/92) | ✓ | ✓ | ✓ | Before Tx |  | 8.4 years*^b^* |  | NS | NS |  |  |  | Carcinoembryonic antigen, N stage, T stage, adjuvant therapy, serum amyloid A |
| Giessen-Jung et al., 2015, Germany [41] | Stage I – III colon cancer (472: 255/217) | ✓ | ✓ | ✓ | Before Tx |  | 5.9 years*^b^* |  | NS | NS |  |  |  | Age, carcinoembryonic antigen, N stage, T stage, CA 19-9, gamma-glutamyl transpeptidase |
| Gwenzi et al., 2023, Germany [77] | Stage I – IV CRC (2819: 1683/1136) | ✓ |  | ✓ | After Tx | rs11568820 | 10 years*^b^* | ⊕ 25(OH) D;  NS (VDR SNP) | ⊕ 25(OH) D;  NS (VDR SNP) | ⊕ 25(OH) D;  NS (VDR SNP) |  | ⊕ 25(OH) D;  NS (VDR SNP) |  | Age, sex, season, cancer stage, tumour site, tumour detection mode, chemotherapy, cardiovascular disease, diabetes, hypertension, smoking, body mass index, physical activity, late entry |
| Hamada et al., 2018, U.S. [52] | Stage I – IV CRC (869: 415/454) |  |  |  | Before Dx, predicted post-diagnostic levels |  | 13.3 years*^b^* | NS | NS |  |  |  |  | Age, sex, year of diagnosis, family history of CRC, prediagnosis predicted 25(OH)D score, tumour site, tumour differentiation, disease stage, microsatellite instability status, CpG island methylator phenotype-specific promoter status, long interspersed nucleotide element-1 methylation level, KRAS mutation, BRAF mutation, PIK3CA mutation, PTGS2 (cyclooxygenase-2) expression |
| Heath et al., 2020, Australia [63] | CRC patients (3065) |  |  |  | ✓ |  | 13.7 years*^a^* |  | ⊕ |  |  |  |  | Age, sex, country of birth, socioeconomic disadvantage, educational attainment, waist circumference, physical activity, smoking status, alcohol intake, mediterranean diet, energy intake |
| Jacobs et al., 2007, U.S. [30] | Patients with resected colorectal adenoma (568: 367/201) |  |  |  | ✓ |  |  |  |  |  | NS |  |  | Sex, body mass index, number of colonoscopies, previous polyps, season of blood draw |
| Jacobs et al., 2016, U.S. [45] | Patients with resected colorectal adenoma (651: 428/223) |  |  |  | ✓ |  |  |  |  |  | NS |  |  | Age, body mass index, season of blood draw |
| Kim et al., 2021, South Korea [85] | Stage I-III CRC (795: 429/366) | ✓ |  | ✓ | Before/ after Tx |  | 36.7 months*^b^* |  |  | ⊕ |  |  |  | Age, pathological stage |
| Kim et al., 2023, U.S. [78] | Stage I – IV CRC (588: 248/340) |  |  |  | Before Dx |  |  | ⊕ | NS |  |  |  |  | Sex, season of blood collection, body mass index, smoking status, physical activity, cancer stage, primary tumor site, year of  diagnosis |
| Lawler et al., 2023, U.S. [79] | Stage 0 – IV CRC (218: 101/117) |  |  |  | Before Dx |  |  | NS | NS |  |  |  |  | Age, sex, education, income, alcohol consumption, smoking history, CRC screening, obesity, insurance coverage, physical activity, tumor stage |
| Maalmi et al., 2017, Germany [48] | Stage I – IV CRC (2910: 1732/1178) | ✓ |  | ✓ | After Dx |  | 4.8 years for OS; 3.9 years for RFS*^b^* | NS | NS | NS |  | NS |  | Age, sex, season of blood draw, cancer stage at diagnosis, tumor location, tumor detection mode, surgery, chemotherapy, history of cardiovascular diseases, history of diabetes, history of hypertension, smoking, body mass index, physical activity, late entry |
| Markotic et al., 2019, Croatia [55] | Stage I – IV CRC (596: 373/223) | ✓ |  |  | Before/ after Tx |  | 5.9 years*^b^* | NS |  |  |  |  |  | Age, sex, month of sampling, tumor stage, tumor location |
| Messaritakis et al., 2020, Greece [16] | Stage II – IV CRC (397: 246/151) | ✓ | ✓ | ✓ |  | rs7975232, rs1544410, rs2228570, rs731236 | 10 years | ⊖ |  |  |  |  | NS (PFS) | ECOG performance status, tumor grade, TLR polymorphism |
| Messaritakis et al., 2022, Greece [72] | Stage III CRC (132: 78/54) | ✓ |  | ✓ |  | rs7975232, rs1544410, rs2228570, rs731236 |  |  |  | ⊖ (rs7975232) |  |  |  | BRAF mutation, histology, detection of microbial DNA encoding for glutamine synthase of *B. fragilis*, detection of microbial DNA encoding for 5.8S rRNA |
| Messaritakis et al., 2023, Greece [80] | Stage III CRC (237: 151/86) | ✓ |  | ✓ |  | rs7975232, rs1544410, rs2228570, rs731236 |  | ⊕ (rs7975232, rs1544410) |  |  |  |  |  |  |
| Mezawa et al., 2010, Japan [32] | Stage I – IV CRC (257) | ✓ |  | ✓ | Peri-operative |  | 32.4 months*^b^* | ⊕ | NS | NS |  |  |  | Age at diagnosis, sex, month of blood sampling, cancer stage, residual tumor after surgery, time period of surgery, location of tumor, adjuvant chemotherapy, number of lymph nodes with metastasis |
| Morelli et al., 2022, Italy [73] | mCRC (133: 60/73) |  |  | ✓ | Before Tx |  |  | ⊕ |  |  |  |  |  | Time since diagnosis, resection of the primary, primary location, synchronous vs metachronous metastasis onset, liver vs. not-liver metastasis, RAS/BRAF status, KPS, age, sex, body mass index, hemoglobin, platelet, monocyte, lymphocyte, neutrophil, lymph/ white blood cell, neutrophil/ white blood cell, neutrophil-lymphocyte ratio, platelet-lymphocyte ratio, systemic inflammatory index, d-dimer, carcinoembryonic antigen, CA19.9, creatinine, alanine aminotransferase, aspartate aminotransferase, alkaline phosphatase, total bilirubin, gamma glutamyl transferase, C-reactive protein, glycemia, albumin, lactate dehydrogenase |
| Ng et al., 2008, U.S. [6] | Stage I – IV CRC (304: 145/159) | ✓ |  | ✓ | Before Dx |  | 78 months*^b^* | ⊕ | NS |  |  |  |  | Age at diagnosis, season of blood draw, sex, cancer stage, grade of tumor differentiation, location of primary tumor, year of diagnosis, body mass index at diagnosis, postdiagnosis physical activity |
| Ng et al., 2011, Canada and U.S. [33] | mCRC (515: 306/209) |  |  | ✓ | Before Tx |  | 5.1 years*^b^* | NS |  |  |  |  | NS (TTP); NS (TR) | Age, season of blood collection, sex, baseline performance status, treatment arm, body mass index, metastatic sites |
| Obermannova et al., 2015, Czech Republic [42] | mCRC (84: 49/35) | ✓ | ✓ | ✓ | ✓ |  | 24.2 months*^b^* | ⊕ |  |  |  |  | ⊕ (PFS) | Carcinoembryonic antigen, any surgical procedure |
| Pérez-Durán et al., 2023, Spain [17] | Stage I – IV CRC (127: 85/42) | ✓ |  | ✓ |  | rs7975232, rs1544410, rs2228570, rs731236, rs11568820 | 35.8 months*^b^* | ⊖ (rs7975232) |  |  |  |  | NS (PFS) for rs7975232 and rs2228570 | Metastasis, age of diagnosis, stage, ECOG score, lymph node involvement, adjuvant chemotherapy, no family history of CRC |
| Perna et al., 2013, Germany [37] | Stage 0 – IV CRC (1397: 852/545) |  |  |  |  | rs2228570, rs731236, rs11568820, rs1989969 | 5 years | NS | NS |  |  |  |  | Age, sex, stage, free from stroke, diabetes mellitus, myocardial infarction |
| Robsahm et al., 2019, Norway [56] | Colon cancer (37: 23/14) |  |  |  | Before Dx and at the time of Dx |  |  |  | ⊕ |  |  |  |  | Age at diagnosis, sex, season of serum sampling, serum storage time |
| Sinicrope et al., 2021, U.S. [69] | Stage III colon cancer (600: 316/284) |  |  | ✓ | Post-surgical |  |  | NS |  | NS | NS |  |  | Treatment arm, race, body mass index, tumor-infiltrating lymphocytes, N stage |
| Slattery et al., 2014, U.S. [39] | Colon cancer (1553: 868/685); Rectal cancer (754: 451/303) |  |  |  |  | *BsmI*, *FokI*, Poly | > 5 years |  | ⊖ colon cancer survival (*BsmI*, Poly) |  |  |  |  | Age, study center, race/ ethnicity, sex, AJCC stage, tumor molecular phenotype |
| Torfadottir et al., 2019, Iceland [57] | CRC (118) |  |  |  | Before Dx |  | 8.3 years*^a^* | ⊕ | NS |  |  |  |  | Age at diagnosis, sex, body mass index, education, smoking status, alcohol intake, physical activity, season of blood sampling |
| Tretli et al., 2012, Norway [35] | Colon cancer (52: 32/20) |  |  |  | After Dx |  |  | NS | NS |  |  |  |  | Age at diagnosis, sex, season of blood sampling |
| Vaughan-Shaw et al., 2020, UK. [64] | Stage I – IV CRC (2006: 1137/869) in time point analysis, (1687: 970/717) in survival analysis in cohort 1; Stage I – IV CRC (2100: 1172/928) in time point analysis, (1848: 1024/824) in survival analysis in cohort 2 | ✓ |  |  | Peri-operative | rs11568820 | 13.3 years*^b^* in cohort 1; 3.6 years*^b^* in cohort 2 | ⊕ | ⊕ |  |  |  |  | Age, sex, AJCC stage, body mass index, tumour site, time between definitive treatment and sampling |
| Väyrynen et al., 2016, Finland [46] | Stage I – IV CRC (117: 58/59) | ✓ |  |  | Before Tx |  | 60 months | NS | NS | NS |  |  |  |  |
| Wang et al., 2023, U.S. [86] | Stage III colon cancer (1437: 795/642) |  |  | ✓ | Before Tx |  | 5.8 years*^b^* | ⊕ |  | ⊕ | ⊕ |  |  | Age, sex, race, body mass index, season, ECOG status, tumor location, tumor stage (T1/2, T3/4), nodal stage, treatment |
| Weinstein et al., 2018, Finland [53] | CRC (497 men) |  |  |  | Before Dx |  | 28 years |  | NS |  |  |  |  | Age at diagnosis, body mass index, number of cigarettes smoked per day, years of smoking, physical activity, serum cholesterol, history of diabetes, family history of cancer, systolic blood pressure, trial intervention group (alpha-tocopherol or beta-carotene), year of diagnosis, prior cancer diagnoses |
| Weinstein et al., 2022, U.S. [74] | CRC (476) |  |  |  | Before Dx |  | 15.6 years*^b^* |  | NS |  |  |  |  | Age at cancer diagnosis, body mass index, smoking status, physical activity, history of diabetes, family history of cancer, year of diagnosis, cancer stage, cancer grade, cancer site |
| Wesa et al., 2015, U.S. [43] | mCRC (250) |  |  | ✓ | Before Tx |  | 41 months*^b^* | ⊕ |  |  |  |  |  | Albumin, ECOG performance status |
| Wesselink et al., 2020, The Netherlands [65] | Stage I – III CRC (1169: 751/418) |  |  |  | Before Tx |  | 3.5 years for recurrence; 4.7 years for OS*^b^* | NS |  |  | NS |  |  | Age, sex, stage, body mass index, physical activity, tumor location, season of blood collection, cohort, total energy intake, total magnesium and calcium intake |
| Wesselink et al., 2021, The Netherlands [70] | Stage I – III CRC (679: 440/239) | ✓ | ✓ | ✓ | After Dx |  | 2.2 years for recurrence; 3.5 years for OS*^b^* | ⊕ |  |  | NS |  |  | Age at diagnosis, sex, stage of disease, cohort, mean magnesium intake over 2 years |
| Xia et al., 2022, China [75] | CRC (112: 70/42) | ✓ |  | ✓ | Before/ 24-hr after Sx |  |  |  |  |  |  |  | ⊖ Surgical site infection; ⊖ Postoperative nausea/ vomiting; ⊖ Pain sensitivity |  |
| Yang et al., 2017, China [49] | Stage I – III CRC (206: 131/75) | ✓ |  | ✓ | Before Sx |  | 45 months*^b^* | NS |  |  |  |  |  | Age, smoking, body mass index, hypertension, diabetes, tumor stage, cell differentiation, albumin |
| Yuan et al., 2019 May, Canada and U.S. [59] | mCRC (524: 309/215) |  |  | ✓ | ✓ | ✓ | 9.2 years*^b^* | ⊕ (25 (OH) D) |  |  |  |  |  | Age, sex, race/ ethnicity, ECOG performance status, no. of metastatic sites, treatment arm, season of blood collection |
| Yuan et al., 2019, Canada and U.S. [58] | Locally advanced or mCRC (1041: 604/437) |  |  | ✓ | After Dx |  | 5.6 years*^b^* | ⊕ |  |  |  |  | ⊕ (PFS) | Age, sex, race, ECOG performance status, prior adjuvant chemotherapy, chemotherapy backbone, assigned treatment arm, RAS mutation status, body mass index, physical activity, season of blood collection, geographic region of residence |
| Yuan et al., 2020, U.S. [66] | Stage I – IV CRC (603: 246/357) |  |  |  | Before Dx |  | 12.4 years*^b^* | NS | ⊕ |  |  |  |  | Age at diagnosis, season of blood collection, sex, body mass index, physical activity, cancer stage, grade of tumor differentiation, location of primary tumor, year of diagnosis, vitamin D binding protein levels |
| Zgaga et al., 2014, UK [40] | Stage I – III CRC (1598) | ✓ |  | ✓ | After Sx | rs7975232, rs1544410, rs10735810, rs11568820 | 8.9 years for overall and 9.6 years for those alive at censor date*^b^* | ⊕ | ⊕ |  |  |  |  | Tumor site, surgery, time between definitive treatment and sampling, season of blood collection, body mass index, physical activity |
| Zhang et al., 2024, UK [81] | Stage I – IV CRC (2936: 1697/1239) in SOCCS cohort; Stage I – IV CRC (3181: 1872/1309) in UKBB cohort |  |  |  | ✓ | rs11568820 | 6.93 years*^a^* in SOCCS cohort; 6.19 years*^a^* in UKBB cohort | ⊕ 25 (OH) D; NS (VDR SNP) | ⊕ 25 (OH) D, NS (VDR SNP) in SOCCS cohort; NS in UKBB cohort |  |  |  |  | Age, sex, tumor site, season of blood sampling, AJCC stage, body mass index |
| Zhou et al., 2021, UK [71] | CRC (2509: 1494/1015) |  |  |  | Before Dx |  | 7.1 years*^b^* | ⊕ | NS |  |  |  |  | Age at diagnosis, sex, ethnicity, fasting status, time interval between assessment and diagnosis of CRC, Townsend deprivation index, college or university degree, body mass index, total physical activity, smoking status, alcohol consumption, family history of CRC, prevalent diabetes, prevalent hypertension, history of cardiovascular diseases, location of primary tumor, histology of tumor, menopausal status (in women), ever use of hormone replacement therapy |
| Zhu et al., 2017, Canada [50] | Stage I – IV CRC (531: 330/201) | ✓ | ✓ | ✓ |  | rs1544410, rs10735810, rs731236 | 6.4 years*^b^* | NS |  | NS |  |  |  | Age at diagnosis, sex, stage at diagnosis, race, reported screening procedure, marital status, MSI status, BRAF mutation status |
| Zhu et al., 2019, Australia [60] | CRC (110: 55/55) |  |  |  | Before Dx |  | 20 years |  | NS |  |  |  |  | Age, sex, season of blood collection, taking vitamin D supplement, marital status, occupation, smoking, alcohol consumption, physical activity, body mass index, diabetes, aspirin use |

CMT, chemotherapy; CPS-II, Cancer Prevention Study-II; CRC, colorectal cancer; CSS, colorectal cancer-specific survival; DFS, disease-free survival; Dx, diagnosis; EPIC, European Prospective Investigation into Cancer and Nutrition; NS, not significant; PFS, progression-free survival; Rec, recurrence; RT, radiotherapy; SNP, single nucleotide polymorphism; SOCCS, Study of Colorectal Cancer in Scotland; Sx, surgery; TR, tumor response; TTR, time to recurrence; TTP, time to progression; Tx, treatment; UKBB, UK Biobank; VDR, vitamin D receptor. ^a^mean, ^b^median; ⊕, increased survival as the increase in vitamin D levels; ⊖, decreased survival

Symbols (⊕, ⊖, NS) reflect the direction and statistical significance of hazard ratios (HRs) and 95% confidence intervals used in the meta-analysis (see **Supplementary Table S6**). For studies included only in the qualitative systematic review, symbols reflect the original study’s reported findings.
