# Supplementary material for: Circulating vitamin D status and prognosis in colorectal cancer: a systematic review and meta-analysis with exploratory evidence on vitamin D receptor polymorphisms
Source: BMC Cancer. 2026 Apr 16;26:687. doi: 10.1186/s12885-026-16026-x (PMC13220566; doi:10.1186/s12885-026-16026-x)
Supplement: Supplementary file 6 — Supplementary Material 6. [file 12885_2026_16026_MOESM6_ESM.docx]

**Supplementary Table S6** Studies included in meta-analysis with specific circulating 25(OH)D categorization

| **Study reference** | **Timing of vitamin D measurement** | **Vitamin D definition** | **Categorization method (Quartile/ clinical cut-offs)** | **Effect estimates used for pooling in meta-analysis** | **Outcome** |
| --- | --- | --- | --- | --- | --- |
| Abrahamsson et al., 2019 [82] | Pre-treatment | Low (<50 nmol/L) vs. High (≥50 nmol/L) | Clinical cut-off | 0.07 (0.01–0.60)^*^ | OS |
| Bao et al., 2020 [61] | Before surgery | High vs. Low | Median-based cut-off (47.50 nmol/L) | 0.54 (0.33–0.88) (Primary cohort) | OS |
|  |  |  |  | 0.22 (0.08–0.60) (Validation cohort) |  |
|  |  |  |  | 0.56 (0.32–0.99) (Primary cohort-stage III subgroup) |  |
| Boakye et al., 2021 [67] | Before or shortly after surgery and before chemo(radio)therapy initiation | Q5 vs. Q1 | z-score quintiles | 0.76 (0.57–1.01) | CSS |
|  |  |  |  | 0.66 (0.52–0.83) | OS |
| Cooney et al., 2013 [36] | Post-diagnosis | T3 (>26.6 ng/mL) vs. T1 (<19 ng/mL); Q5 (>30.8 ng/mL) vs. Q1 (<15.5 ng/mL) | Tertiles; Quintiles | 1.01 (0.59–1.73) | CSS |
|  |  |  |  | 1.06 (0.64–1.75) | OS |
| Dolin et al., 2023 [76] | Before surgery | Deficient (<25 nmol/L) vs. Sufficient (>50 nmol/L) | Clinical cut-off | 0.29 (0.11–0.80)^*^ | OS |
| Facciorusso et al., 2016 [44] | Post-diagnosis | >20 ng/mL vs. ≤20 ng/mL | Clinical cut-off | 0.35 (0.21–0.59) | OS |
|  |  |  |  | 0.37 (0.17–0.80) | TTR |
| Fedirko et al., 2012 [34] | Pre-diagnosis | Q5 (>76.8 nmol/L) vs. Q1 (<36.3 nmol/L) | Quintiles | 0.69 (0.51–0.94) | CSS |
|  |  |  |  | 0.67 (0.51–0.89) | OS |
| Fuchs et al., 2017 [47] | Pre-diagnosis | Q5 (31.5 ng/mL^§^) vs. Q1 (23.3 ng/mL^§^) | Quintiles | 0.55 (0.38–0.80) | OS |
|  |  |  |  | 0.65 (0.46–0.92) | RFS |
|  |  |  |  | 0.62 (0.44–0.87) | DFS |
| Gibbs et al., 2020 [62] | NR | Deficient (<30 nmol/L) vs. Sufficient (>50 nmol/L) | Clinical cut-off | 0.75 (0.58–0.97)^*^ | CSS |
|  |  |  |  | 0.73 (0.59–0.92)^*^ | OS |
| Gwenzi et al., 2023 77] | Post-operative | Sufficient (≥50 nmol/L) vs. Deficient (<30 nmol/L) | Clinical cut-off | 0.76 (0.60–0.96) | CSS |
|  |  |  |  | 0.71 (0.60–0.85) | OS |
|  |  |  |  | 0.79 (0.64–0.98) | RFS |
|  |  |  |  | 0.69 (0.58–0.82) | DFS |
| Heath et al., 2020 [63] | NR | Q4 (Women 53.1–121.3, Men 68.9–201.8 nmol/L) vs. Q1 (Women 13.9–34.7, Men 8.2–43.0 nmol/L) | Quartiles | 0.60 (0.36–0.99) | CSS |
| Kim et al., 2021 [85] | At diagnosis (baseline), and at 6-, 12-, and 18-month post-surgery | ≥12 ng/mL vs. <12 ng/mL | Clinical cut-off | 0.56 (0.35–0.91) | DFS |
| Kim et al., 2023 [78] | Pre-diagnosis | Deficient (<30 nmol/L) vs. Sufficient (>50 nmol/L) | Clinical cut-off | 0.66 (0.33–1.34)^*^ | CSS |
|  |  |  |  | 0.48 (0.31–0.75)^*^ | OS |
| Lawler et al., 2023 [79] | Pre-diagnosis | Sufficient (≥20 ng/mL) vs. Deficient (<12 ng/mL) | Clinical cut-off | 0.83 (0.41–1.70) | CSS |
|  |  |  |  | 0.61 (0.37–1.01) | OS |
| Maalmi et al., 2017 [48] | Post-diagnosis | Deficient (<30 nmol/L) vs. Sufficient (>50 nmol/L) | Clinical cut-off | 0.79 (0.60–1.04)^*^ | CSS |
|  |  |  |  | 0.79 (0.63–1.00)^*^ | OS |
|  |  |  |  | 0.93 (0.73–1.18)^*^ | RFS |
|  |  |  |  | 0.89 (0.72–1.11)^*^ | DFS |
| Markotic et al., 2019 [55] | Before/ after surgery | Sufficient (>50 nmol/L) vs. Deficient (≤50 nmol/L) | Clinical cut-off | 0.86 (0.65–1.14) | OS |
| Morelli et al., 2022 [73] | Pre-treatment | <10 ng/mL vs. >10 ng/mL | Clinical cut-off | 0.49 (0.31–0.78)^*^ | OS |
| Ng et al., 2008 [6] | Pre-diagnosis | Q4 vs. Q1 | Quartiles | 0.61 (0.31–1.20) | CSS |
|  |  |  |  | 0.52 (0.29–0.94) | OS |
| Ng et al., 2011 [33] | Pre-treatment | Q4 (27.2-75.4 ng/mL) vs. Q1 (2.3-13.1 ng/mL) | Quartiles | 0.94 (0.72–1.23) | OS |
| Robsahm et al., 2019 [56] | Pre-diagnosis and at the time of diagnosis | Category 4 (>77 nmol/L) vs. Category 1 (≤44 nmol/L) | Clinical cut-off | 0.18 (0.03–1.03) | CSS |
| Tretli et al., 2012 [35] | Post-diagnosis | Q4 (>77 nmol/L) vs. Q1 (≤44 nmol/L) for CSS; Q4 (>81 nmol/L) vs. Q1 (<46 nmol/L) for OS | Quartiles | 0.20 (0.04–1.05) | CSS |
|  |  |  |  | 0.40 (0.10–1.60) | OS |
| Vaughan-Shaw et al., 2020 [64] | Peri-operative | T3 (>33.1 nmol/L) vs. T1 (<18.1 nmol/L) for cohort 1; T3 (>57.9 nmol/L) vs. T1 (<38.0 nmol/L) for cohort 2 | Tertiles | 0.71 (0.55–0.92) (Cohort 1) | CSS |
|  |  |  |  | 0.62 (0.40–0.96) (Cohort 2) |  |
|  |  |  |  | 0.69 (0.56–0.85) (Cohort 1) | OS |
|  |  |  |  | 0.63 (0.44–0.90) (Cohort 2) |  |
| Wang et al., 2023 [86] | Pre-treatment | Non-deficient (≥12 ng/mL) vs. Deficient (<12 ng/mL) | Clinical cut-off | 0.57 (0.40–0.81) | OS |
|  |  |  |  | 0.71 (0.52–0.97) | TTR |
|  |  |  |  | 0.68 (0.51–0.91) | DFS |
| Weinstein et al., 2018 [53] | Pre-diagnosis | Q5 vs. Q1 | Quantiles | 0.96 (0.61–1.50) | CSS |
| Weinstein et al., 2022 [74] | Pre-diagnosis | Q5 vs. Q1 | Quantiles | 1.48 (0.81–2.70) | CSS |
| Wesa et al., 2015 [43] | Pre-treatment | Adequate (≥30 ng/mL) vs. Deficient (<30 ng/mL) | Clinical cut-off | 0.61 (0.38–0.98) | OS |
| Wesselink et al., 2020 [65] | Pre-treatment | Severely deficient (<30 nmol/L) vs. Sufficient (50–74 nmol/L) | Clinical cut-off | 0.72 (0.45–1.15)^*^ | OS |
|  |  |  |  | 0.84 (0.49–1.45)^*^ | TTR |
| Wesselink et al., 2021 [70] | Post-diagnosis | Consistently sufficient (>50 nmol/L) vs. Consistently deficient (<50 nmol/L) | Clinical cut-off | 0.39 (0.21–0.73) | OS |
|  |  |  |  | 0.40 (0.14–1.17) | TTR |
| Yang et al., 2017 [49] | Pre-operative | T3 (>1.03 ng/mL) vs. T1 (<0.58 ng/mL) | Tertiles | 0.40 (0.08–1.93) | OS |
| Yuan et al., 2019 [58] | Post-diagnosis | Q5 (27.5 ng/mL^§^) vs. Q1 (8.0 ng/mL^§^) | Quintiles | 0.66 (0.53–0.83) | OS |
| Yuan et al., 2020 [66] | Pre-diagnosis | Q4 (383.5 μg/mL^¥^) vs. Q1 (125.2 μg/mL^¥^) | Quartiles | 0.57 (0.34–0.96) | CSS |
|  |  |  |  | 0.72 (0.49–1.05) | OS |
| Zgaga et al., 2014 [40] | Post-operative | T3 (>13.25 ng/mL) vs. T1 (<7.25 ng/mL) | Tertiles | 0.68 (0.51–0.91) | CSS |
|  |  |  |  | 0.70 (0.55–0.89) | OS |
| Zhang et al., 2024 [81] | NR | Group 3 (>50 nmol/L) vs. Group 1 (<25 nmol/L) | Clinical cut-off | 0.45 (0.32–0.64) (SOCCS) | CSS |
|  |  |  |  | 0.83 (0.67–1.03) (UKBB) |  |
|  |  |  |  | 0.60 (0.46–0.78) (SOCCS) | OS |
|  |  |  |  | 0.72 (0.60–0.86) (UKBB) |  |
| Zhou et al., 2021 [71] | Pre-diagnosis | Q4 (≥60.85 nmol/L) vs. Q1 (<34.35 nmol/L) | Quartiles | 0.85 (0.66–1.10) | CSS |
|  |  |  |  | 0.80 (0.65–0.99) | OS |
| Zhu 2019 [60] | Pre-diagnosis | Higher (≥75 nmol/L) vs. Lower (<50 nmol/L) | Clinical cut-off | 0.99 (0.56–1.75) | CSS |

CSS, Colorectal cancer-specific survival; DFS, disease-free survival; NR, Not reported; OS, overall survival; TTR, time to recurrence

**Note:** Effect estimates were harmonized to represent high versus low vitamin D levels. For studies reporting effect estimates as low versus high, hazard ratios (HRs) and corresponding 95% confidence intervals (CIs) were inverted for inclusion in the meta-analysis (high vs. low). For some studies, minor discrepancies between reported and pooled effect estimates are due to rounding during transformation and meta-analysis computations.

^*^ For studies reporting effect estimates as low versus high, hazard ratios were inverted, and the corresponding 95% confidence intervals were recalculated accordingly.

^§^ Median

^¥^ Mean
