# Supplementary material for: Circulating vitamin D status and prognosis in colorectal cancer: a systematic review and meta-analysis with exploratory evidence on vitamin D receptor polymorphisms
Source: BMC Cancer. 2026 Apr 16;26:687. doi: 10.1186/s12885-026-16026-x (PMC13220566; doi:10.1186/s12885-026-16026-x)
Supplement: Supplementary file 7 — Supplementary Material 7. [file 12885_2026_16026_MOESM7_ESM.docx]

**Supplementary Table S7** Ongoing registered clinical trials investigating vitamin D or VDR in colorectal cancer patients^a^

| **NCT number** | **Country** | **Title** | **Population** | **Intervention** | **Vitamin D/ VDR** | **Outcome measures** |
| --- | --- | --- | --- | --- | --- | --- |
| NCT04094688 | U.S. | Vitamin D3 with chemotherapy and bevacizumab in treating patients with advanced or metastatic colorectal cancer (SOLARIS) | Metastatic CRC | Arm I: Bevacizumab, chemotherapy, high-dose vitamin D3  Arm II: Bevacizumab, chemotherapy, standard-dose vitamin D3 | 25(OH)D levels | PFS, treatment response, OS, adverse events |
| NCT02172651 | U.S. | Vitamin D in patients with stage I-III colon cancer or resectable colon cancer liver metastases | Stage I-III colon cancer | Oral vitamin D3 | 25(OH)D levels, VDR | VDR binding sites, TME changes, adverse events |
| NCT01570010 | Norway | The Norwegian dietary guidelines and colorectal cancer survival study (CRC-NORDIET) | Stage I-III CRC | Dietary intervention | 25(OH)D levels | DFS, OS, TTR, CSS, biomarkers |
| NCT02912559 | U.S. | Combination chemotherapy with or without atezolizumab in treating patients with stage III colon cancer and deficient DNA mismatch repair | Stage III colon cancer | Arm I (combination chemotherapy, atezolizumab)  Arm II (combination chemotherapy) | 25(OH)D levels | DFS, OS, adverse events |

^a^, information retrieved from ClinicalTrials.gov, accessed March 2026; CRC, colorectal cancer; CSS, CRC-specific survival; DFS, disease-free survival; OS, overall survival; PFS, progression-free survival; TME, tumor microenvironment; TTR, time to recurrence; VDR, vitamin D receptor
