# Supplementary material for: Circulating vitamin D status and prognosis in colorectal cancer: a systematic review and meta-analysis with exploratory evidence on vitamin D receptor polymorphisms
Source: BMC Cancer. 2026 Apr 16;26:687. doi: 10.1186/s12885-026-16026-x (PMC13220566; doi:10.1186/s12885-026-16026-x)
Supplement: Supplementary file 8 — Supplementary Material 8. [file 12885_2026_16026_MOESM8_ESM.docx]

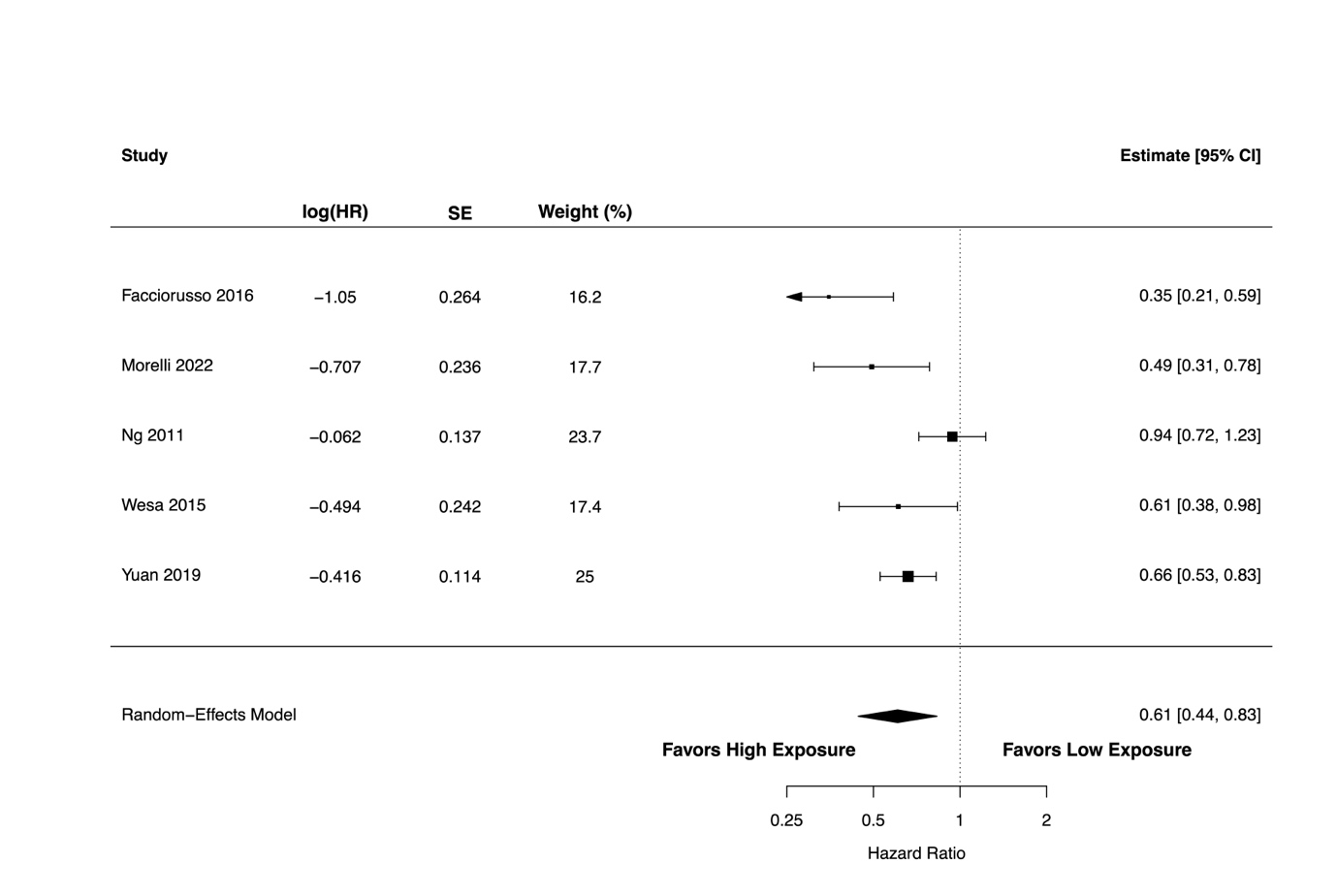


**Supplementary Figure S1**. A forest plot showing the association between high versus low circulating 25(OH)D levels and overall survival in metastatic colorectal cancer patients.


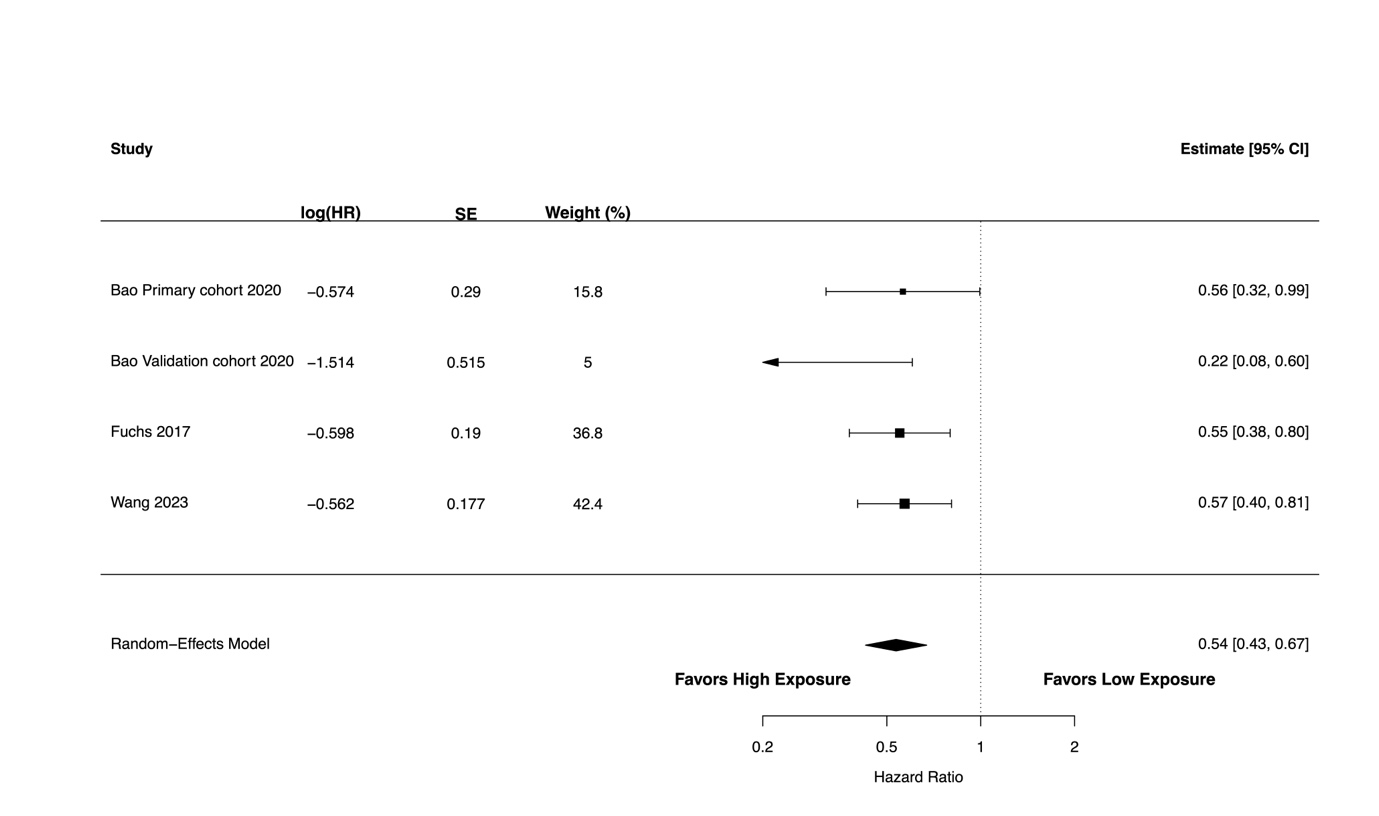


**Supplementary Figure S2**. A forest plot showing the association between high versus low circulating 25(OH)D levels and overall survival in stage III colorectal cancer patients.


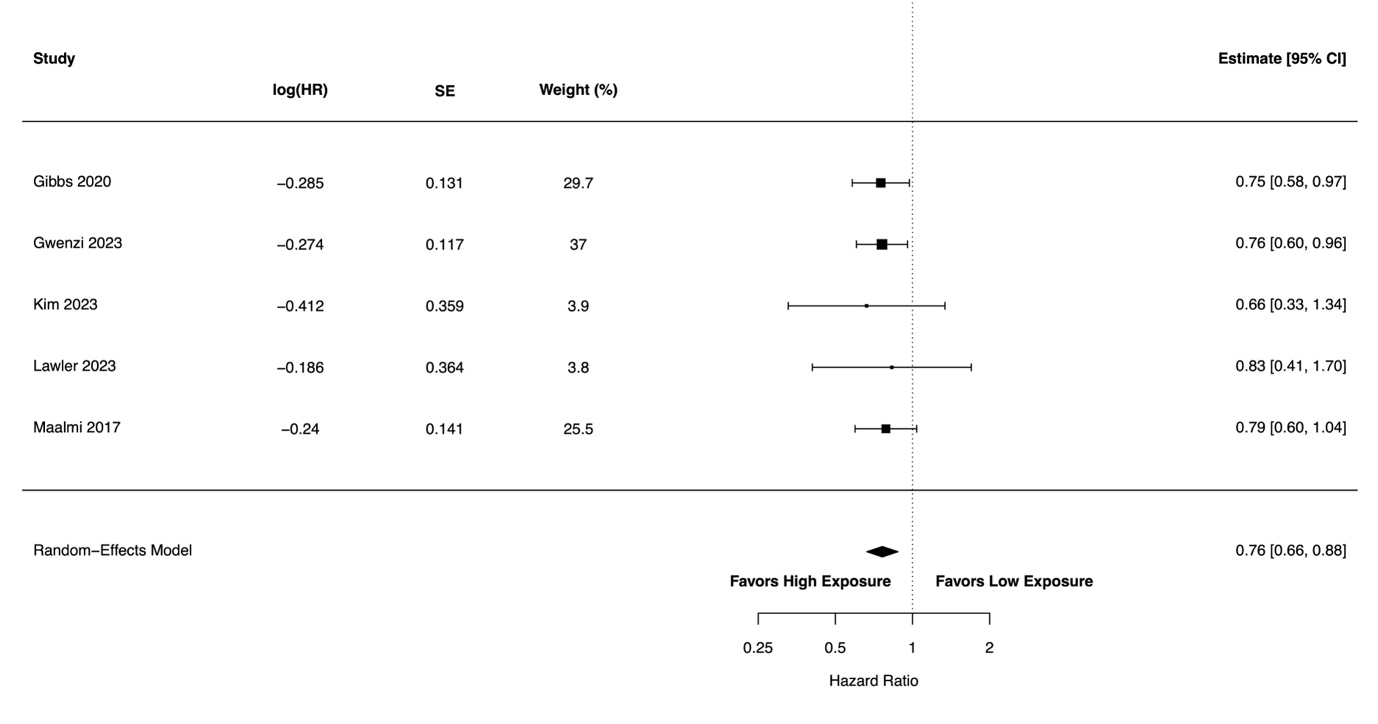


**Supplementary Figure S3**. A forest plot showing the association between high versus low circulating 25(OH)D levels and colorectal cancer-specific survival, from a sensitivity analysis restricted to studies with comparable vitamin D thresholds (≥50 nmol/L or ≥20 ng/mL vs. <30 nmol/L or <12 ng/mL).


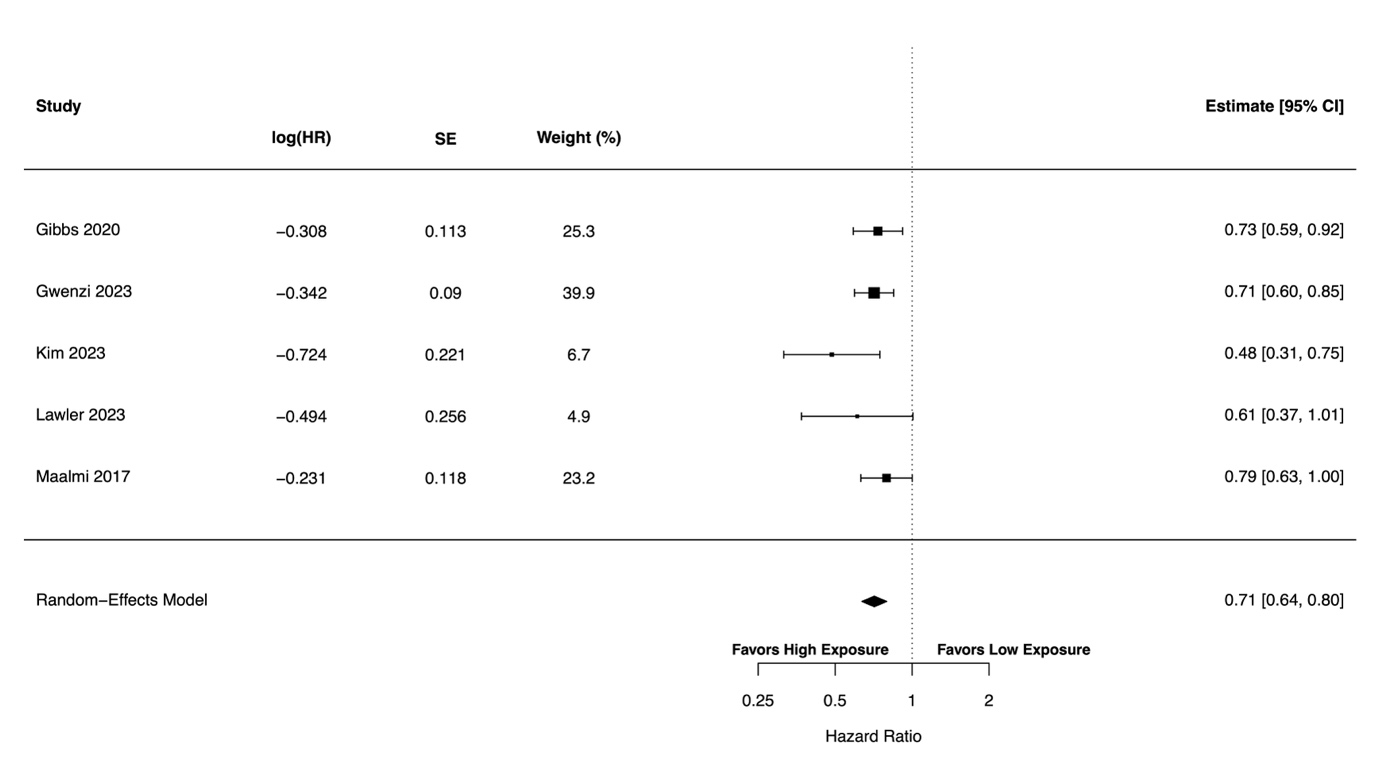


**Supplementary Figure S4**. A forest plot showing the association between high versus low circulating 25(OH)D levels and overall survival, from a sensitivity analysis restricted to studies with comparable vitamin D thresholds (≥50 nmol/L or ≥20 ng/mL vs. <30 nmol/L or <12 ng/mL).


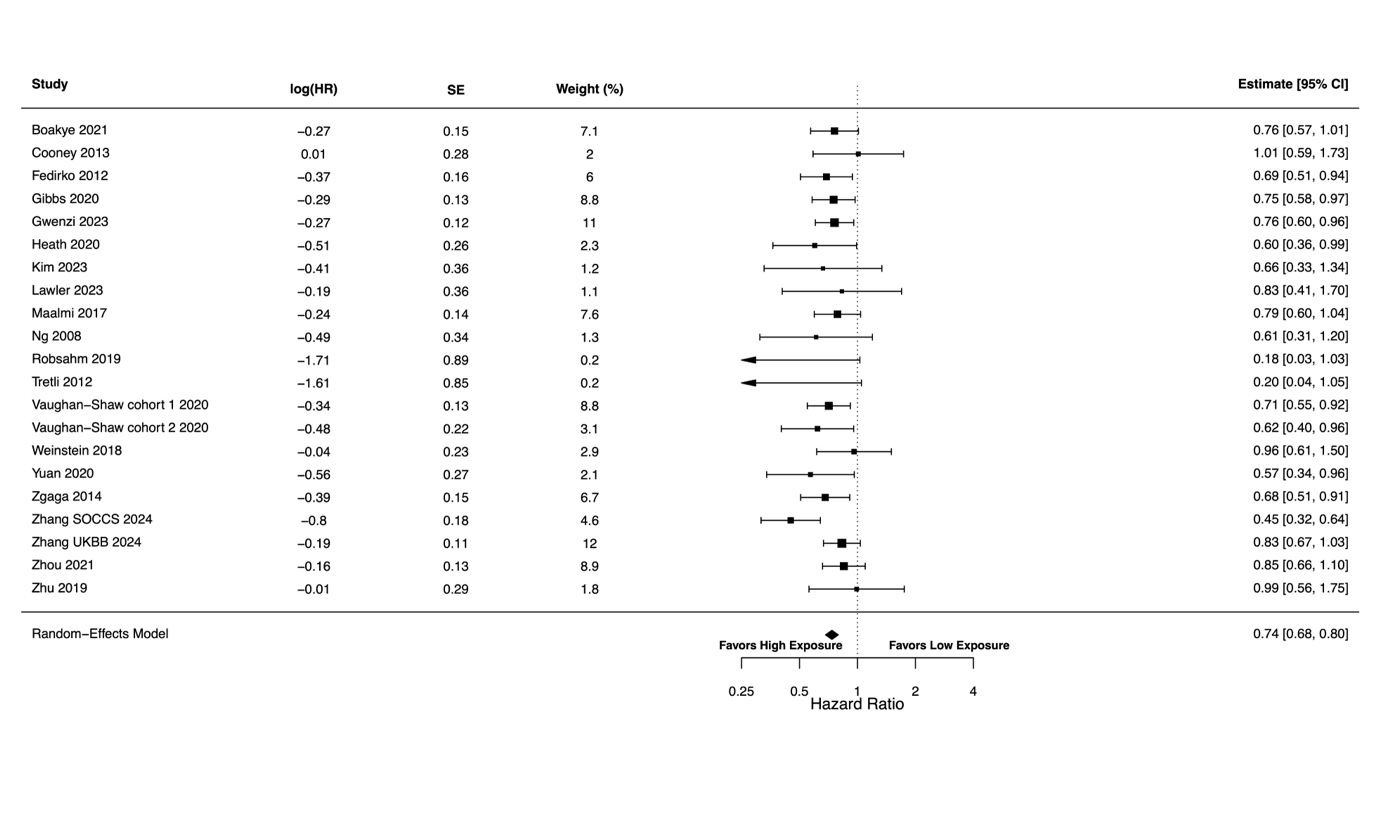


**Supplementary Figure S5**. A forest plot showing the association between high versus low circulating 25(OH)D levels and colorectal cancer-specific survival, from a sensitivity analysis excluding a study rated as low quality.


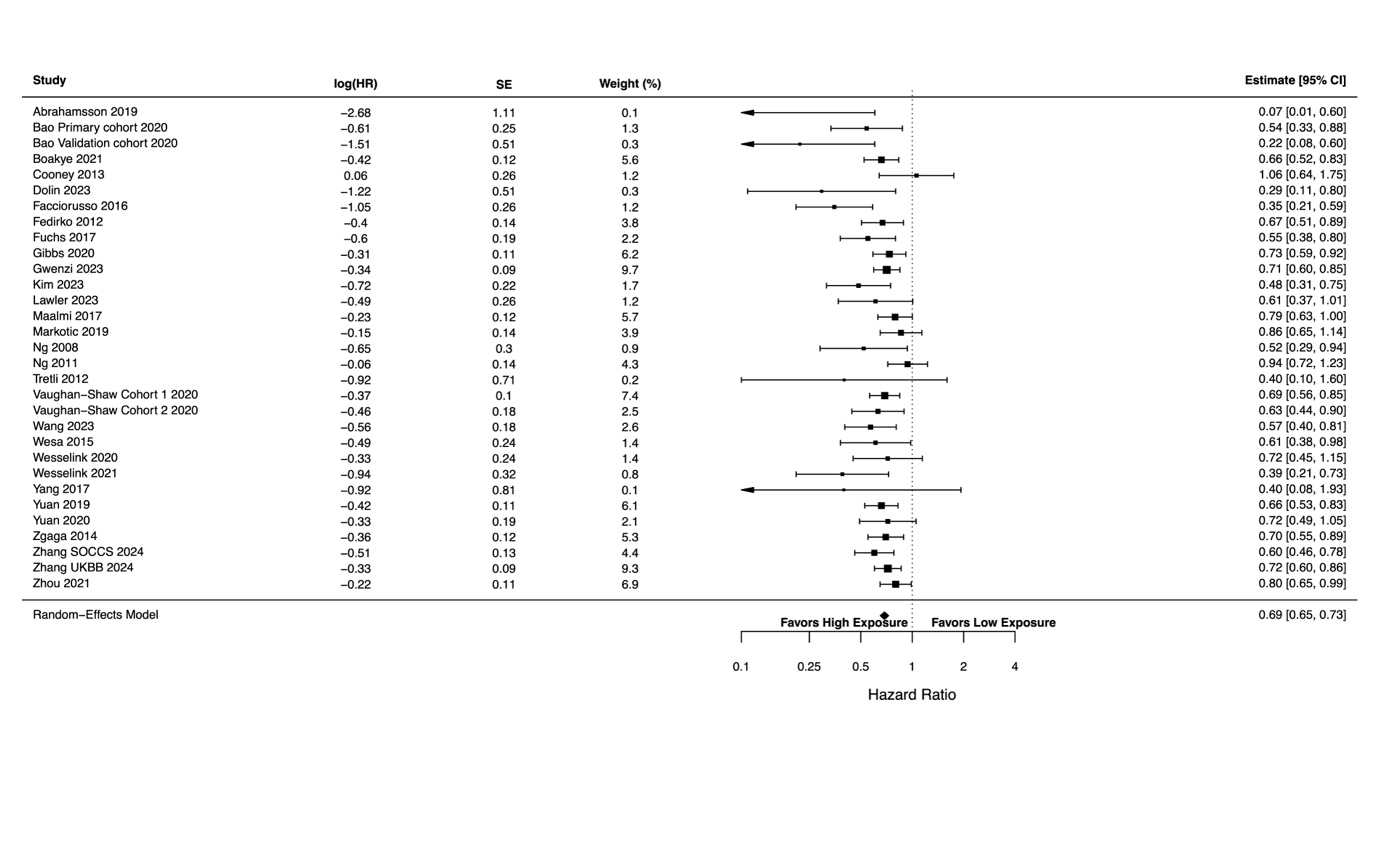


**Supplementary Figure S6**. A forest plot showing the association between high versus low circulating 25(OH)D levels and overall survival, from a sensitivity analysis excluding a study rated as low quality.


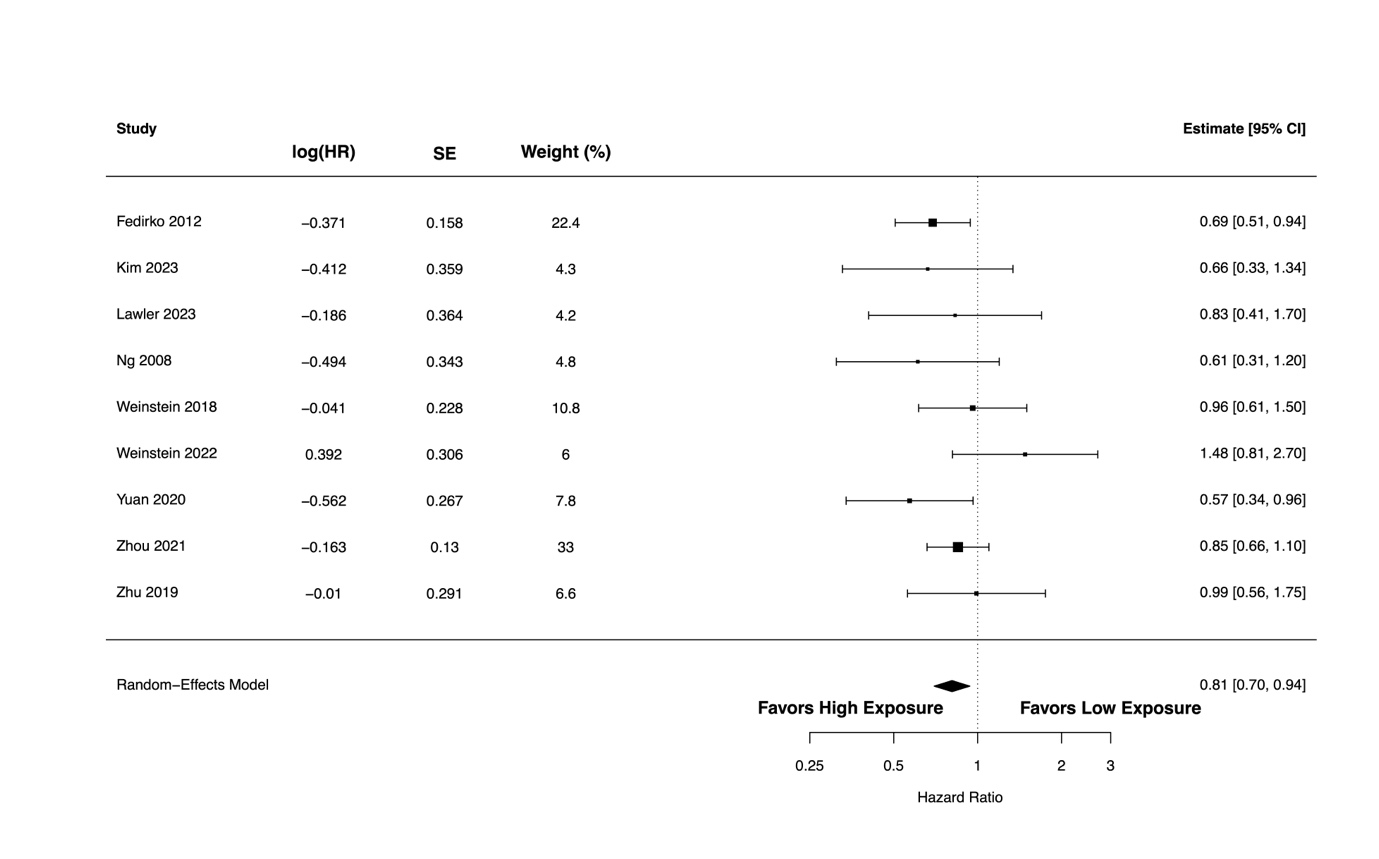


**Supplementary Figure S7**. A forest plot showing the association between high versus low pre-diagnostic circulating 25(OH)D levels and colorectal cancer-specific survival.


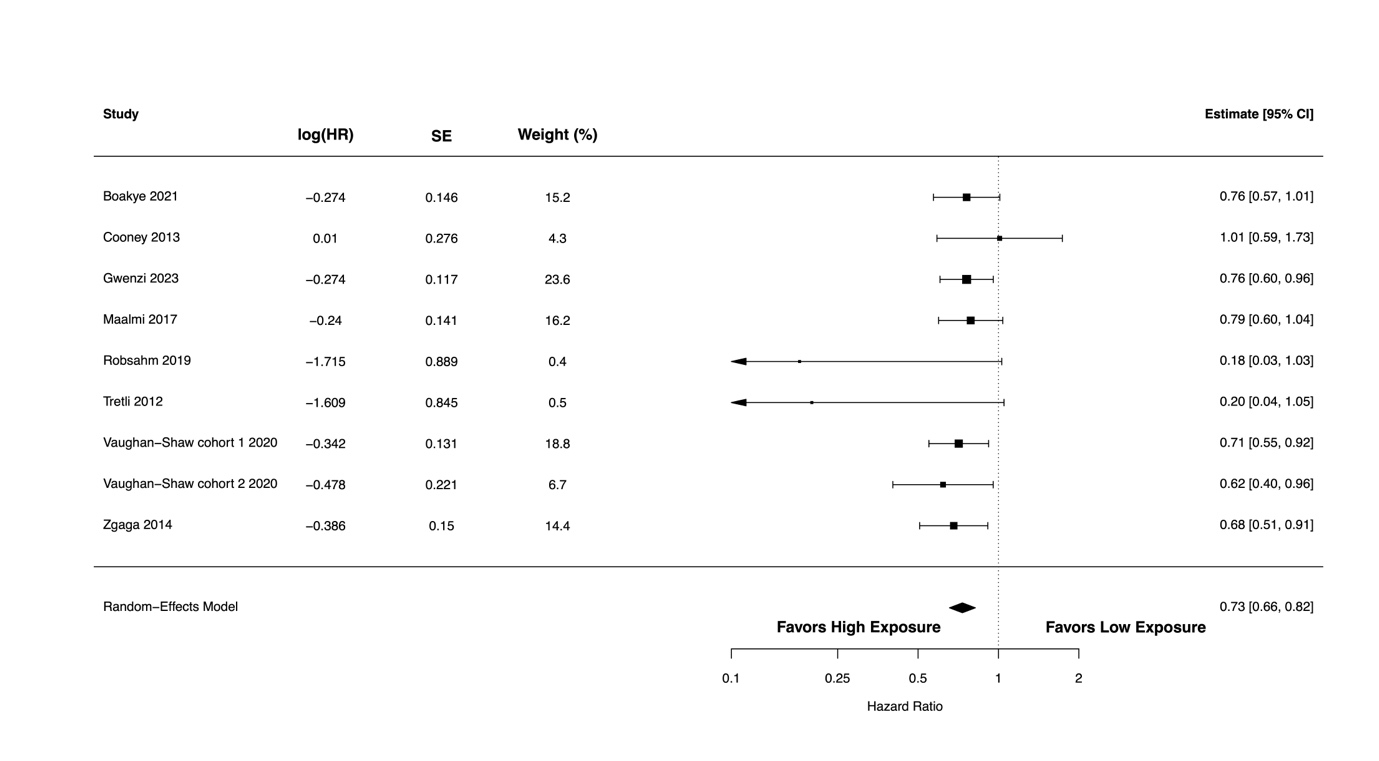


**Supplementary Figure S8**. A forest plot showing the association between high versus low post-diagnostic circulating 25(OH)D levels and colorectal cancer-specific survival.


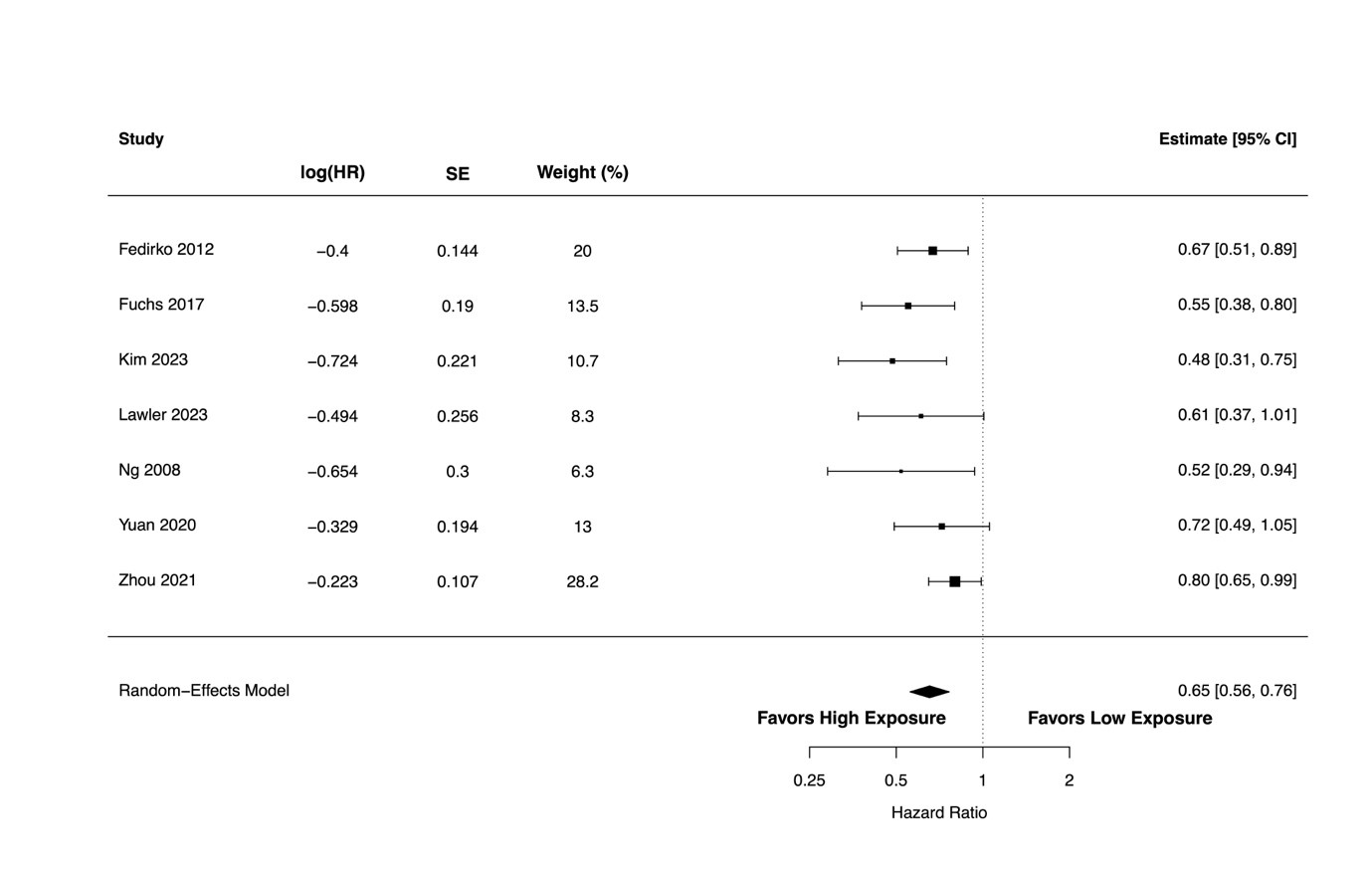


**Supplementary Figure S9**. A forest plot showing the association between high versus low pre-diagnostic circulating 25(OH)D levels and overall survival.


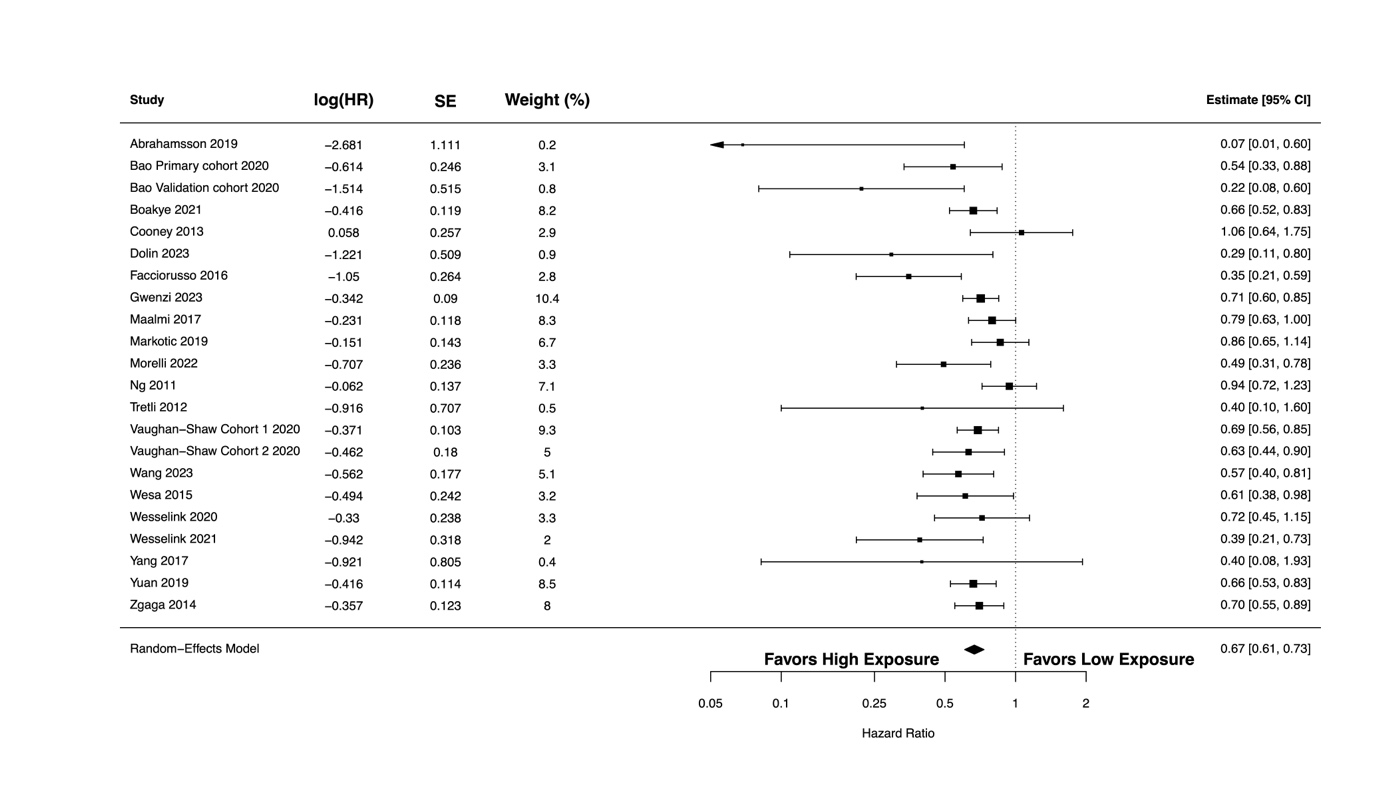


**Supplementary Figure S10**. A forest plot showing the association between high versus low post-diagnostic circulating 25(OH)D levels and overall survival.
